# Phase 1 study of mTORC1/2 inhibitor sapanisertib (TAK-228) in advanced solid tumours, with an expansion phase in renal, endometrial or bladder cancer

**DOI:** 10.1038/s41416-020-01041-x

**Published:** 2020-09-11

**Authors:** Martin H. Voss, Michael S. Gordon, Monica Mita, Brian Rini, Vicky Makker, Teresa Macarulla, David C. Smith, Andrés Cervantes, Igor Puzanov, Roberto Pili, Ding Wang, Shadia Jalal, Shubham Pant, Manish R. Patel, Rachel l. Neuwirth, Aaron Enke, Yaping Shou, Farhad Sedarati, Douglas V. Faller, Howard A. Burris

**Affiliations:** 1 Department of Medicine, 300 East 66th Street, Memorial Sloan Kettering Cancer Center, New York, NY 10065 USA; 2 Oncology Research, HonorHealth Research Institute, 10510 N 92nd St Suite 200, Scottsdale, AZ 85258 USA; 3 Department of Hematology and Oncology, Cedars-Sinai Medical Center, Samuel Oschin Comprehensive Cancer Institute, 8700 Beverly Blvd North Tower, Los Angeles, CA 90048 USA; 4 Cleveland Clinic Foundation, Department of Solid Tumor Oncology, 9500 Euclid Avenue, Cleveland, OH 44195 USA; 5 Medical Oncology Department, Vall d’Hebron University Hospital and Vall d’Hebron Institute of Oncology (VHIO), Passeig de la Vall d’Hebron, 119, 129, 08035 Barcelona, Spain; 6 University of Michigan, Department of Internal Medicine, 1500 E. Medical Center Drive, Ann Arbor, MI 48109 USA; 7 CIBERONC, Department of Medical Oncology, Biomedical Research Institute INCLIVA, University of Valencia, Avda. Menéndez Pelayo 4 acc., 46010 Valencia, Spain; 8 Vanderbilt University Medical Center, Department of Medicine, Division of Hematology/Oncology, 1161 21st Ave S, Nashville, TN 37232 USA; 9 Indiana University—Simon Cancer Center, Department of Medicine, Division of Hematology/Oncology, 535 Barnhill Drive, Indianapolis, IN 46202 USA; 10 Henry Ford Health System, Hematology/Oncology, 1 Ford Pl, Detroit, MI 48202 USA; 11 Indiana University Melvin and Bren Simon Cancer Center, Hematology/Oncology, 535 Barnhill Drive, Indianapolis, IN 46202 USA; 12 Department of Investigational Cancer Therapeutics, The University of Texas MD Anderson Cancer Center, 1515 Holcombe Blvd, Houston, TX 77030 USA; 13 Florida Cancer Specialists/SCRI, Drug Development Unit, 600 N Cattlemen Rd #200, Sarasota, FL 34232 USA; 14 Biostatistics, Millennium Pharmaceuticals, Inc., 40 Landsdowne Street, Cambridge, MA 02139 USA; 15 Oncology Clinical Research, Millennium Pharmaceuticals Inc., 40 Landsdowne Street, Cambridge, MA 02139 USA; 16 Sarah Cannon Research Institute/Tennessee Oncology, Drug Development Unit, 250 25th Ave., North Nashville, TN 37203 USA; 17 Present Address: Clovis Oncology, San Francisco, CA USA; 18 Present Address: Trillium Therapeutics Inc., Cambridge, MA USA

**Keywords:** Urological cancer, Cancer therapy, Gynaecological cancer

## Abstract

**Background:**

This Phase 1 dose-escalation/expansion study assessed safety/tolerability of sapanisertib, an oral, highly selective inhibitor of mTORC1/mTORC2, in advanced solid tumours.

**Methods:**

Eligible patients received increasing sapanisertib doses once daily (QD; 31 patients), once weekly (QW; 30 patients), QD for 3 days on/4 days off QW (QD × 3dQW; 33 patients) or QD for 5 days on/2 days off QW (QD × 5dQW; 22 patients). In expansion cohorts, 82 patients with renal cell carcinoma (RCC), endometrial or bladder cancer received sapanisertib 5 mg QD (39 patients), 40 mg QW (26 patients) or 30 mg QW (17 patients).

**Results:**

Maximum tolerated doses of sapanisertib were 6 mg QD, 40 mg QW, 9 mg QD × 3dQW and 7 mg QD × 5dQW. Frequent dose-limiting toxicities (DLTs) included hyperglycaemia, maculo-papular rash (QD), asthenia and stomatitis (QD × 3dQW/QD × 5dQW); expansion phase doses of 5 mg QD and 30 mg QW were selected based on tolerability beyond the DLT evaluation period. One patient with RCC achieved complete response; nine experienced partial responses (RCC: seven patients; carcinoid tumour/endometrial cancer: one patient each). Sapanisertib pharmacokinetics were time-linear and supported multiple dosing. Pharmacodynamic findings demonstrated treatment-related reductions in TORC1/2 biomarkers.

**Conclusions:**

Sapanisertib demonstrated a manageable safety profile, with preliminary antitumour activity observed in RCC and endometrial cancer.

**Clinical trial registration:**

ClinicalTrials.gov, NCT01058707.

## Background

The phosphoinositide 3-kinase (PI3K)/protein kinase B (AKT)/mammalian target of rapamycin (mTOR) signalling pathway is a central regulator of cellular growth, proliferation and survival.^1^ Dysregulation of PI3K/AKT/mTOR activity is frequently observed in human cancers.^2^ As part of the mammalian target of rapamycin complex 1 (mTORC1) and 2 (mTORC2), mTOR is a key intracellular point of convergence for several pathways, thus representing an important therapeutic target. Inhibition of mTOR may decrease protein translation and prevent abnormal cell proliferation and tumour angiogenesis.^3,4^ Accordingly, rapamycin analogues (“rapalogs”), such as temsirolimus and everolimus, have been approved by the US Food and Drug Administration for the treatment of advanced renal cell cancer (RCC) and several other cancers.^1,5–10^ Rapalogs exert their effect mainly on mTORC1, with only a mild inhibitory effect on mTORC2.^11^ Inhibition of mTORC1, without mTORC2 inhibition, can result in the activation of AKT through a negative feedback mechanism, which may limit rapalog efficacy by accelerating tumour progression.^12^

Preclinical data have demonstrated that inhibition of AKT activity, through mTORC2 inhibition, may block tumour progression.^13^ Subsequently, a new generation of mTORC1/2 inhibitors have been developed, as dual inhibition may offer an advantage over mTORC1 inhibitors by targeting at least three key enzymes (PI3K, AKT and mTOR).^14^ Proof of this concept was demonstrated in preclinical models for several epithelial malignancies.^15–18^

Sapanisertib is an investigational, oral and highly selective adenosine triphosphate-competitive mTOR kinase inhibitor that suppresses both mTORC1 and mTORC2. Preclinical models of efficacy, safety and pharmacokinetics (PK) of sapanisertib have largely relied on daily dosing.^19–23^ Investigation of different dosing schedules in preclinical efficacy models has demonstrated commensurate tumour growth inhibition with administration of sapanisertib in daily or intermittent dosing schedules.^19–23^ These preclinical data generally indicated that efficacy was related to total exposure (i.e. area under the curve) and independent of dosing schedule. However, the clinical tolerability was expected to be different for different schedules. Therefore, we evaluated a range of dosing schedules in this first-in-human Phase 1 study (NCT01058707) of sapanisertib in patients with advanced solid tumours, with an expansion phase including patients with RCC, endometrial or bladder cancer.

## Methods

### Study design

This Phase 1, open-label, dose-escalation and expansion study aimed to determine the safety, tolerability and preliminary efficacy of sapanisertib in patients with advanced solid tumours. During dose-escalation, patients received one of four sapanisertib dosing schedules: once daily (QD), once weekly (QW; days 1, 8, 15 and 22), QD for 3 days on/4 days off each week (QD × 3dQW; days 1–3, 8–10, 15–17 and 22–24) and QD for 5 days on/2 days off each week (QD × 5dQW; days 1–5, 8–12, 15–19 and 22–26) in a 28-day cycle with dose escalation based on a modified Fibonacci schema.

Definitions for dose-limiting toxicities (DLTs), as predetermined in the study protocol, were: any grade ≥3 non-haematologic toxicity (except inadequately treated grade 3 nausea and/or vomiting and grade 3 diarrhoea [all patients should have received optimal antiemetic and/or antidiarrhoeal prophylaxis treatment], grade 3 hyperglycaemia lasting ≤14 days [all patients should have received optimal anti-glycaemic treatment, including insulin) and grade 3 rash lasting ≤3 days [all patients should have received topical steroid treatment, oral antihistamines and pulse oral steroids, if necessary]); grade 4 neutropenia lasting >7 days in the absence of growth factor support; grade 4 neutropenia of any duration accompanied with fever ≥38.5 °C and/or systemic infection; any other grade ≥4 haematologic toxicity.

Sapanisertib dosing was withheld for Grade ≥ 3 treatment-related toxicities. If the event resolved to Grade ≤ 1 or baseline values within 28 days of interrupting therapy, the patient could resume study treatment at a ≥25% dose reduction or, for patients in the dose-escalation phase, at the next lower dose level with the sponsor’s approval. If dose modification was required for subjects receiving ≤4 mg QD, then the dosing frequency was decreased to 5 days per week, instead of decreasing the daily dose administered. If sapanisertib dosing was delayed for >28 consecutive days for treatment-related toxicity, despite supportive treatment per standard clinical practice, or more than 2 dose reductions of sapanisertib were required in a patient, sapanisertib therapy was stopped, the patient was discontinued from the study and the follow-up visit was completed within 30 days of the last administration of sapanisertib.

The maximum tolerated dose (MTD) was to be determined in the QD schedule before enrolment of initial single-patient cohorts for the QW and QDx3d QW schedules. If a grade ≥ 2 AE was observed in any single-patient cohort, an additional 2–5 patients were assigned to that cohort. Subsequent dose cohorts for that schedule included 3–6 patients. Dose cohorts for the QDx5dQW schedule enrolled three patients with an additional three patients enrolled if a DLT was observed per a standard 3 + 3 design. Once the MTD was identified for each of the dosing schedules, an additional six patients were enrolled to obtain further PK and safety data prior to expansion.

Based on data collected during dose-escalation, the expansion phase evaluated the safety and efficacy of QD and QW sapanisertib in patients with RCC, endometrial or bladder cancer. Patients could continue receiving sapanisertib for ≤1 year (or beyond, if the investigator and sponsor agreed) in the absence of disease progression or unacceptable toxicity.

The primary objective was to determine the MTD and DLTs for each sapanisertib dosing schedule and evaluate the safety and tolerability of sapanisertib in both phases. Secondary objectives were to evaluate preliminary antitumour activity, PK in peripheral blood, and pharmacodynamics (PD) of sapanisertib, as measured by modulation in phosphorylation of S6, 4EBP1 and NDRG1 in surrogate tissue (skin) and tumour.

The study was conducted in accordance with the Declaration of Helsinki and Good Clinical Practice. Institutional review boards approved all aspects of the study. All participants provided written informed consent.

### Patients

Eligible patients were aged ≥18 years with locally advanced or metastatic solid tumours who had failed standard-of-care therapy, had an Eastern Cooperative Oncology Group performance status of 0–1, and had adequate bone marrow, hepatic, renal and metabolic (fasting serum glucose ≤ 130 mg/dL and fasting triglycerides ≤ 300 mg/dL) function.

Patients with locally advanced or metastatic brain tumours were eligible if their brain metastases had been treated (without evidence of progression or haemorrhage post-treatment), and if they had not taken dexamethasone 4 weeks prior to the first study drug administration and with no ongoing requirement for dexamethasone or antiepileptic drugs. In the expansion phase, eligible patients had measurable disease per the Response Evaluation Criteria in Solid Tumors (RECIST), v1.1.

Patients could be considered for enrolment in one of three disease-specific cohorts: (1) advanced or recurrent endometrial adenocarcinoma with disease progression following ≥1 prior chemotherapy regimen; (2) advanced/metastatic urothelial cancer (carcinoma of the bladder, ureter and/or renal pelvis), progressive after ≥1 prior therapy in the metastatic/unresectable setting; (3) advanced RCC after failure of ≥1 prior antivascular endothelial growth factor therapy with no prior TORC1 inhibitor therapy, or progressed on treatment with TORC1 inhibitor therapy.

Patients who had received prior cancer therapy within 2 weeks, systemic corticosteroid therapy within 1 week prior to the first dose of study drug, or bisphosphonates within 30 days prior to the first sapanisertib dose were not eligible. Additionally, patients with impaired cardiac function or significant active cardiovascular disease were also excluded. In the expansion phase, patients who had received prior AKT, PI3K, dual PI3K/TORC1/2 or TORC1/2 inhibitors were excluded.

### Assessments

Patients who received ≥1 dose of sapanisertib were included in the safety population. The response-evaluable population included patients who received ≥1 dose of sapanisertib, had measurable disease at baseline, and had ≥1 postbaseline assessment. In the dose-escalation phase, the evaluable population included patients who received ≥75% of the planned sapanisertib doses in cycle 1 or experienced a DLT. Response was assessed according to the RECIST v1.1^24^ after every two treatment cycles. Adverse events (AEs) were assessed using the National Cancer Institute Common Terminology Criteria for Adverse Events, v4.0. Patients were provided with a home blood-glucose meter to monitor their fasting predose blood-glucose measurements to assess hyperglycaemia as an on-target AE and PD marker.

Peripheral blood was collected serially to quantify plasma levels of sapanisertib for PK analysis via validated liquid chromatography tandem mass spectrometry with an assay range of 1–1000 ng/mL (MicroConstants, San Diego, CA), during both dose-escalation and expansion phases. Blood samples were collected before each dose and 0.5, 1, 2, 4, 8 and 24 h after dosing on cycle 1, day 1 and cycle 2, day 1, in the dose-escalation phase. In the expansion phase, samples were collected predose and 2, 4 and 6 h postdose on cycle 1, day 1, and then predose and 2 h postdose on day 8, 15 or 22 of cycle 1.

The tissue PK and PD of sapanisertib were investigated in skin and tumour biopsies. During dose escalation, 3-mm core skin biopsies were collected predose and on any day from 8–15 (3 h postdose) of cycle 1 for immunohistochemistry (IHC) assessment of mTORC1 downstream effects (p4EBP1, pS6) and pNDRG1 (Mosaic Laboratories, Lake Forest, CA). IHC for p-AKT did not pass validation and is not reported. Archival tumour tissues for assessment of prognostic markers were collected at baseline.

Statistical analyses were primarily descriptive and graphical in nature, with no formal statistical hypothesis testing.

## Results

### Patients

From December 2009 to January 2013, 116 patients were enrolled to the dose-escalation phase and received single-agent sapanisertib in the following schedules: QD (*n* = 31), QW (*n* = 30), QD × 3dQW (*n* = 33) and QD × 5dQW (*n* = 22). From March 2013 to April 2014, an additional 82 patients with RCC, endothelial or bladder cancer were enrolled in the expansion phase to receive sapanisertib 5 mg QD (*n* = 39), 40 mg QW (*n* = 26) or 30 mg QW (*n* = 17). Baseline demographics and characteristics are shown in Table 1.

**Table 1:** Patient baseline characteristics and demographics.

	Sapanisertib								
	Dose-escalation phase					Expansion phase			
Characteristic	QD 2–7 mg (*n* = 31)	QD × 5dQW 7–13 mg (*n* = 22)	QD × 3dQW 6–20 mg (*n* = 33)	QW 7–40 mg (*n* = 30)	Total (*n* = 116)	QD 5 mg (*n* = 39)	QW 30 mg (*n* = 17)	QW 40 mg (*n* = 26)	Total (*n* = 82)
Median age, years (range)	61 (24–75)	62 (32–75)	54 (36–87)	57 (34–89)	60 (24–89)	61 (30–81)	63 (32–76)	65 (44–80)	62 (30–81)
Male, *n* (%)	15 (48)	9 (41)	11 (33)	12 (40)	47 (41)	23 (59)	11 (65)	12 (46)	46 (56)
Race, *n* (%)									
Asian	1 (3)	0	0	0	1 (1)	1 (3)	0	0	1 (1)
White	28 (90)	22 (100)	30 (91)	29 (97)	109 (94)	37 (95)	17 (100)	25 (96)	79 (96)
Cancer diagnosis, *n* (%)									
Breast	3 (10)	1 (5)	2 (6)	2 (7)	8 (7)	–	–	–	–
Colorectal	6 (19)	8 (36)	7 (21)	4 (13)	25 (22)	–	–	–	–
Gastric	0	0	1 (3)	1 (3)	2 (2)	–	–	–	–
Head and neck	1 (3)	1 (5)	2 (6)	1 (3)	5 (4)	–	–	–	–
Lung (non-small-cell)	0	1 (5)	4 (12)	3 (10)	8 (7)	–	–	–	–
Melanoma	1 (3)	0	0	0	1 (1)	–	–	–	–
Ovarian	2 (6)	1 (5)	4 (12)	2 (7)	9 (8)	–	–	–	–
Pancreatic	2 (6)	0	2 (6)	1 (3)	5 (4)	–	–	–	–
Prostate	1 (3)	0	1 (3)	0	2 (2)	–	–	–	–
Renal	2 (6)	3 (14)	1 (3)	4 (13)	10 (9)	22 (56)	10 (59)	13 (50)	45 (55)
RCC, TORC1i naïve	–	–	–	–	–	8 (21)	4 (24)	8 (31)	20 (24)
RCC, TORC1i failure	–	–	–	–	–	14 (36)	6 (35)	5 (19)	25 (30)
Endometrial	3 (10)	0	1 (3)	4 (13)	8 (7)	11 (28)	4 (24)	6 (23)	21 (26)
Bladder	–	–	–	–	–	6 (15)	3 (18)	6 (23)	15 (18)
Other	–	–	–	–	–	–	–	1 (4)^a^	1 (1)
Number of prior treatment regimens, median (range)	3 (1–10)	2 (0–5)	4 (0–6)	3 (0–10)	3 (0–10)	2 (0–7)	2 (0–4)	2 (0–8)	2 (0–8)

*QD* once daily, *QD* × *3dQW* QD for 3 days on/4 days off QW, *QD* × *5dQW* QD for 5 days on/2 days off QW, *QW* once weekly, *TORC1i* target of rapamycin complex 1 inhibitor therapy.

^a^Patient initially diagnosed with metastatic transitional cell carcinoma of the renal pelvis; re-review of the patient’s pathology slides revealed primary renal urothelial carcinoma.

### DLTs and MTD determination

Dose escalation, DLTs and MTDs are summarised in Table 2. The MTD was determined to be 6 mg for the QD schedule based on 4/10 patients reporting a DLT in cycle 1 (grade 3 maculo-papular rash [*n* = 1], grade 3 diarrhoea [*n* = 1], grade 3 asthenia [*n* = 1], grade 5 ventricular fibrillation/cardiac arrest [*n* = 1]) and 40 mg for the QW schedule based on 2/12 patients reporting a DLT (grade 3 dry mouth and fatigue [*n* = 1], grade 3 asthenia [*n* = 1]), 9 mg for the QD × 3dQW schedule based on 1/6 evaluable patients reporting a DLT (grade 3 hypophosphatemia) and 7 mg for the QD × 5dQW schedule based on all three patients receiving 13 mg reporting DLTs (grade 3 fatigue, asthenia and stomatitis [*n* = 1 each]), 4/13 patients receiving 10 mg reporting DLTs (grade 2 stomatitis [*n* = 1], grade 3 stomatitis [*n* = 1], grade 3 asthenia [*n* = 1], grade 3 stomatitis and fatigue [*n* = 1]) and no patients reported DLTs at 7 mg.

**Table 2:** Dose escalation and determination of maximum tolerated dose (MTD).

	Treated patients^a^, *n*	Evaluable patients^b^, *n*	Patients with DLTs, *n*	DLTs
QD dosing schedule				
2 mg	3	3	0	–
4 mg	7	7	1	Grade 3 hyperglycaemia
6 mg^c^ (MTD)	13	10	4	Grade 3 maculo-papular rash; grade 3 diarrhoea; grade 3 asthenia; grade 5 ventricular fibrillation/cardiac arrest
7 mg	8	5	2	Grade 3 hyperglycaemia and grade 4 anaemia; grade 3 maculo-papular rash
QW dosing schedule				
7 mg	3	3	0	–
10 mg	3	3	0	–
15 mg	3	3	0	–
20 mg	3	3	0	–
30 mg	3	3	0	–
40 mg (MTD)	15	12	2	Grade 3 dry mouth and fatigue; grade 3 asthenia
QD × 3dQW dosing schedule				
6 mg	3	3	0	–
9 mg (MTD)	8	6	1	Grade 3 hypophosphatemia
12 mg	6	6	2	Grade 3 stomatitis and grade 3 dehydration; grade 3 asthenia
16 mg	12	11^d^	1	Grade 3 stomatitis
20 mg	4	3	2	Grade 3 stomatitis; grade 3 stomatitis
QD × 5dQW dosing schedule				
7 mg (MTD)	6	6	0	–
10 mg	13	13	4	Grade 2 stomatitis; grade 3 stomatitis; grade 3 asthenia; grade 3 stomatitis; grade 3 fatigue
13 mg	3	3	3	Grade 3 fatigue; grade 3 asthenia; grade 3 stomatitis

*AE* adverse event, *DLT* dose-limiting toxicity, *QD* once daily, *QD* × *3dQW* once daily for 3 days on and 4 days off each week, *QD* × *5dQW* once daily for 5 days on and 2 days off each week, *QW* once weekly.

^a^Initial dose cohorts for each of the alternate dosing schedules prior to a protocol amendment enrolled a single patient. If grade ≥ 2 AE, regardless of relatedness to sapanisertib was observed in any single-patient cohort, an additional 2–5 patients were assigned to that cohort and subsequent dose cohorts in that treatment arm would include 3–6 patients.

^b^Patients who received ≥75% of the planned doses of sapanisertib in cycle 1 or stopped study drug before receiving 75% of the planned doses because of a study treatment-related AE considered a DLT.

^c^Patients were enrolled into the 6 mg QD dosing schedule after the 7 mg QD dosing schedule.

^d^Five patients required dose modification due to AEs.

Based on biochemical, PK and tolerability data, and early signs of antitumour activity, the QD and QW schedules were selected for expansion. Sapanisertib 5 mg QD was selected rather than the MTD of 6 mg, because the 6 mg dose was poorly tolerated beyond the DLT evaluation period in 10 evaluable patients. The QW 40 mg dose was determined as the MTD based on DLT criteria and was initially selected for expansion. However, follow-up of the first 21 patients enrolled at this dose revealed that several patients required dose modifications or discontinued treatment due to AEs; the recommended expansion dose was thereafter reduced to 30 mg QW.

### Treatment exposure and safety

During dose escalation, patients received a median of two treatment cycles (range, 1–58; Supplementary Table 1). Across all doses, 97% of patients experienced treatment-related AEs (TRAEs), and 11% experienced at least one treatment-related grade ≥ 3 AE (Supplementary Table 1). The most common TRAEs were hyperglycaemia, nausea, stomatitis, vomiting, decreased appetite and diarrhoea (Table 3). Hyperglycaemia was the most common grade ≥ 3 TRAE (Supplementary Table 2), which was generally well controlled with metformin and home glucose monitoring. Serious AEs occurred in 43% of patients; 24% discontinued treatment due to an AE (Supplementary Table 1).

**Table 3:** Treatment-related adverse events (AEs) by preferred term reported in ≥15% of patients by dosing schedule.

	Sapanisertib								
	Dose-escalation phase					Expansion phase			
AE, *n* (%)	QD 2–7 mg (*n* = 31)	QD × 5dQW 7–13 mg (*n* = 22)	QD × 3dQW 6–20 mg (*n* = 33)	QW 7–40 mg (*n* = 30)	Total (*n* = 116)	QD 5 mg (*n* = 39)	QW 30 mg (*n* = 17)	QW 40 mg (*n* = 26)	Total (*n* = 82)
Hyperglycaemia	25 (81)	11 (50)	22 (67)	17 (57)	75 (65)	17 (44)	12 (71)	20 (77)	49 (60)
Nausea	13 (42)	13 (59)	23 (70)	22 (73)	71 (61)	20 (51)	11 (65)	20 (77)	51 (62)
Stomatitis^a^	11 (35)	13 (59)	23 (70)	9 (30)	56 (48)	19 (49)	8 (47)	10 (38)	37 (45)
Vomiting	8 (26)	10 (45)	18 (55)	17 (57)	53 (46)	12 (31)	10 (59)	19 (73)	41 (50)
Decreased appetite	11 (35)	7 (32)	17 (52)	8 (27)	43 (37)	17 (44)	6 (35)	14 (54)	37 (45)
Diarrhoea	11 (35)	8 (36)	13 (39)	9 (30)	41 (35)	17 (44)	5 (29)	13 (50)	35 (43)
Asthenia	6 (19)	9 (41)	10 (30)	12 (40)	37 (32)	7 (18)	1 (6)	4 (15)	12 (15)
Fatigue	8 (26)	7 (32)	12 (36)	8 (27)	35 (30)	22 (56)	11 (65)	23 (88)	56 (68)
Rash	8 (26)	3 (14)	7 (21)	2 (7)	20 (17)	1 (3)	0 (0)	1 (4)	2 (2)
Dysgeusia	8 (26)	4 (18)	4 (12)	3 (10)	19 (16)	10 (26)	3 (18)	3 (12)	16 (20)
Pruritus generalised	8 (26)	1 (5)	6 (18)	0 (0)	15 (13)	11 (28)	2 (12)	2 (8)	15 (18)
Blood creatinine increased	7 (23)	0 (0)	2 (6)	3 (10)	12 (10)	3 (8)	1 (6)	4 (15)	8 (10)
Dry mouth	5 (16)	2 (9)	2 (6)	3 (10)	12 (10)	3 (8)	1 (6)	6 (23)	10 (12)
Hypercholesterolemia	5 (16)	1 (5)	4 (12)	1 (3)	11 (9)	0 (0)	1 (6)	0 (0)	1 (1)
Pruritus	2 (6)	3 (14)	1 (3)	2 (7)	8 (7)	7 (18)	1 (6)	3 (12)	11 (13)
Hypophosphatemia	0 (0)	2 (9)	5 (15)	0 (0)	7 (6)	3 (8)	2 (12)	4 (15)	9 (11)
Weight decreased	1 (3)	2 (9)	2 (6)	2 (7)	7 (6)	5 (13)	0 (0)	4 (15)	9 (11)
Headache	0 (0)	1 (5)	3 (9)	2 (7)	6 (5)	0 (0)	0 (0)	4 (15)	4 (5)
Rash maculo-papular	3 (10)	2 (9)	1 (3)	0 (0)	6 (5)	14 (36)	2 (12)	1 (4)	17 (21)
Anaemia	3 (10)	0 (0)	1 (3)	1 (3)	5 (4)	2 (5)	2 (12)	8 (31)	12 (15)
Dehydration	3 (10)	0 (0)	1 (3)	1 (3)	5 (4)	2 (5)	2 (12)	5 (19)	9 (11)
Acute kidney injury	1 (3)	0 (0)	0 (0)	0 (0)	1 (1)	2 (5)	0 (0)	4 (15)	6 (7)
Dyspepsia	1 (3)	0 (0)	1 (3)	1 (3)	3 (3)	1 (3)	3 (18)	2 (8)	6 (7)

*QD* once daily, *QD* × *3dQW* once daily for 3 days on and 4 days off each week, *QD* × *5dQW* once daily for 5 days on and 2 days off each week, *QW* once weekly.

^a^Includes oropharyngeal pain and mucosal inflammation.

During expansion, patients received a median of two treatment cycles (range, 1–26; Supplementary Table 1). All patients in this phase experienced at least one TRAE; 46% experienced at least one grade ≥ 3 TRAE (Supplementary Table 1). TRAEs and all-cause AEs in the expansion phase are summarised in Table 3 and Supplementary Table 3, respectively. The most common TRAEs according to schedule were fatigue (56%) and nausea (51%) at 5 mg QD, fatigue (88%), nausea and hyperglycaemia (77% each) at 40 mg QW, and hyperglycaemia (71%), nausea and fatigue (65% each) at 30 mg QW (Table 3). Supplementary Table 2 and Supplementary Table 4 summarise grade ≥ 3 TRAEs and all-cause AEs, respectively, in the expansion phase. Hyperglycaemia was also the most common grade ≥ 3 TRAE in the expansion phase. Serious AEs occurred in 41% of patients and 15% discontinued due to an AE.

AEs resulting in treatment discontinuation during the expansion phase occurred in 18% of patients receiving 5 mg QD or 30 mg QW dosing and 8% of patients receiving 40 mg QW dosing. Fewer patients in the 30 mg QW dose group had their dose modified or reduced than in the other dose groups (47% for 30 mg QW vs 69% for 5 mg QD and 77% for 40 mg QW) (Supplementary Table 1).

There were 7 on-study deaths within 30 days of last dose. Four patients died during dose-escalation: 1 due to ventricular fibrillation and cardiac arrest (6 mg QD considered possibly related to sapanisertib by the investigator, in a patient with pre-existing cardiovascular risk factors), 3 from progressive cancer; 2 gastric (10 mg QW [*n* = 1], 9 mg QD × 3dQW [*n* = 1]) and 1 breast (12 mg QD × 3dQW). Three patients died during expansion due to disease progression (30 mg QW [*n* = 2], 40 mg QW [*n* = 1]).

### Antitumour activity

At data cut-off (02 October 2015), two patients, one with RCC receiving 15 mg QW (dose escalation; 58 cycles) and one with TORC1 inhibitor−naïve RCC receiving 30 mg QW (expansion; 26 cycles), were still receiving study drug. Tumour response across both study phases is summarised in Table 4 and Supplementary Fig. 1. Supplementary Fig. 1 also shows study duration and best percentage change for the expansion phase. During dose-escalation, two patients with RCC receiving 15 mg QW and 40 mg QW achieved a partial response (PR) maintained for >32 months for an objective response rate (ORR) of 8%, and one with a carcinoid tumour receiving 10 mg QD × 5dQW achieved a PR maintained for > 32 months for an ORR of 6%. Five patients in dose-escalation maintained stable disease (SD) for ≥ 6 months. In the expansion phase, one patient with TORC1 inhibitor−naïve RCC receiving 40 mg QW achieved a complete response (CR) maintained for 16 months. Five additional patients with RCC achieved PRs: three TORC1 inhibitor–naïve (5 mg QD, 30 mg QW, and 40 mg QW) for an ORR of 22%, and two with TORC1 inhibitor failure (both 5 mg QD) for an ORR of 9%. There was also one PR in a patient with endometrial cancer receiving 5 mg QD (ORR of 6%). Four of the seven objective responses reported in the expansion phase persisted for > 16 months: three patients with TORC1 inhibitor–naïve RCC (30 mg QW [*n* = 1], 40 mg QW [*n* = 2]) and one with TORC1 inhibitor failure RCC (5 mg QD). Six patients with RCC maintained SD for ≥6 months. Among patients with bladder cancer (*n* = 13), no objective responses were achieved, and 1/5 patients with SD maintained SD for ≥ 6 months.

**Table 4:** Tumour response according to RECIST v1.1 (investigator assessment) in response-evaluable patients.

	Sapanisertib							
	Dose-escalation phase				Expansion phase			
	QD 2–7 mg (*n* = 21)	QD × 5d 7–13 mg (*n* = 18)	QD × 3d 6–20 mg (*n* = 27)	QW 7–40 mg (*n* = 25)	RCC TORC1 naïve (*n* = 18)	RCC TORC1 failure (*n* = 23)	Endometrial cancer (*n* = 18)^a^	Bladder cancer (*n* = 13)
Best overall response, *n* (%)								
CR	0	0	0	0	1 (6)	0	0	0
PR	0	1 (6)	0	2 (8)	3 (17)	2 (9)	1 (6)	0
SD ≥ 6 months	2 (10)	1 (6)	1 (4)	1 (4)	1 (6)	5 (22)	0	1 (8)
SD < 6 months	9 (43)	4 (22)	12 (44)	9 (36)	7 (39)	9 (39)	8 (44)	4 (31)
PD	10 (48)	12 (67)	14 (52)	13 (52)	5 (28)	7 (30)	8 (44)	8 (62)
ORR (CR + PR)	0	1 (6)	0	2 (8)	4 (22)	2 (9)	1 (6)	0
CBR (CR + PR + SD ≥ 6 months)	2 (10)	2 (11)	1 (4)	3 (12)	5 (28)	7 (30)	1 (6)	1 (8)

*CBR* clinical benefit rate, *CR* complete response, *ORR* overall response rate, *PD* progressive disease, *PR* partial response, *QD* once daily, *QD* × *3dQW* once daily for 3 days on and 4 days off each week, *QD* × *5dQW* once daily for 5 days on and 2 days off each week, *QW* once weekly, *RCC* renal cell carcinoma, *RECIST* Response Evaluation Criteria in Solid Tumors, *SD* stable disease, *TORC1* target of rapamycin complex 1.

^a^One patient included in response-evaluable population but response not recorded.

### Pharmacokinetics

**Fig. 1:**
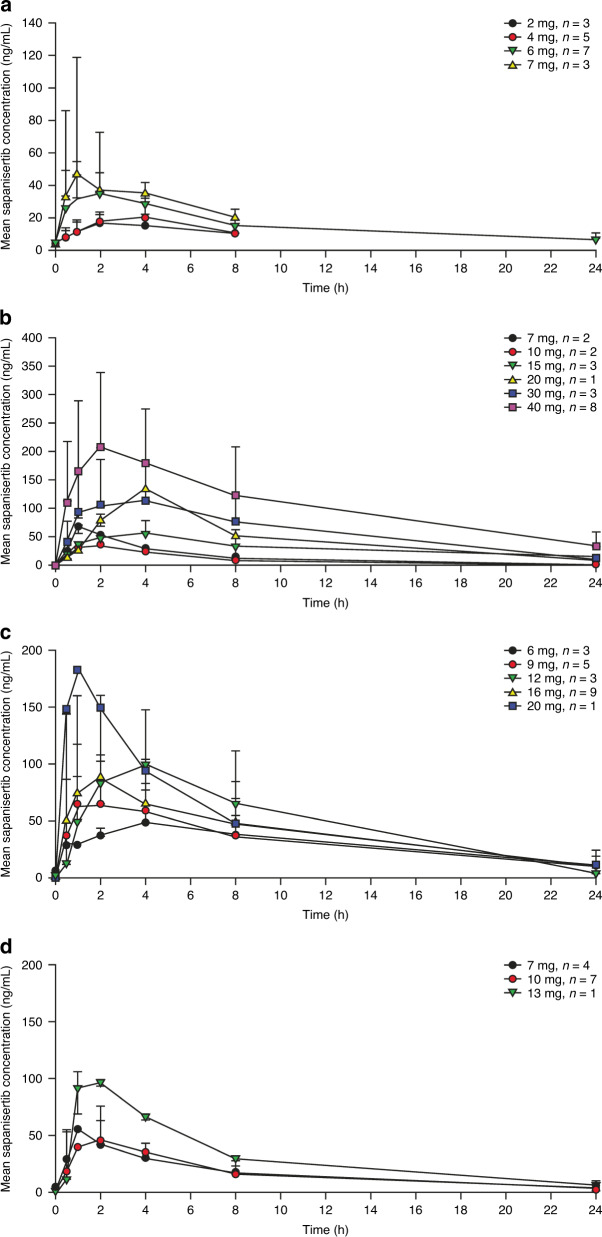
Sapanisertib pharmacokinetics are dose dependent without plasma accumulation over time. Mean (SD) plasma concentration–time profiles of multiple-dose sapanisertib (cycle 2, day 1) on the **a** QD, **b** QW, **c** QD × 3dQW and **d** QD × 5dQW dosing schedules. Error bars indicate SD. *QD* once daily, *QD* × *3dQW* QD for 3 days on/4 days off QW, *QD* × *5dQW* QD for 5 days on/2 days off QW, *QW* once weekly, *SD* standard deviation.

Cycle 1, day 1, PK data were available from 112 patients and cycle 2, day 1, PK data were available from 70 patients. Single-dose (cycle 1, day 1) and multiple-dose (cycle 2, day 1) PK data are summarised in Fig. 1, Supplementary Fig. 2 and Supplementary Table 5. Sapanisertib exhibited rapid oral absorption, with a median T_max_ ranging from 1.0 − 2.8 h across all doses (Supplementary Table 5). Sapanisertib plasma concentrations generally increased in a dose-dependent manner within the 2–40 mg dose range; mean plasma half-life ranged from 5.9 to 9.4 h, with PK variability percentage coefficient of variation (%CV) values for area under the curve from time zero to 24 h postdose (AUC_0–24h_) ranging from ~26 to 87%. Sapanisertib did not accumulate in plasma to any appreciable extent with repeat dosing in any of the schedules (Fig. 1). Sapanisertib PK were generally consistent during both assessment periods (cycle 1, day 1, and cycle 2, day 1), indicating a lack of a time-dependent accumulation with repeat dosing.

### Pharmacodynamics

During dose-escalation, the PD effect of sapanisertib on downstream effectors of TORC1 (p4EBP1 and pS6) and TORC2 (pPRAS40 and pNDRG1) was measured in skin-tissue biopsies from 88 patients collected at baseline and days 8–15 (3 h postdose) of cycle 1. Treatment-related decreases in p4EBP1, pS6, pPRAS40 and pNDRG1 were consistent with dual TORC1/2 inhibition at sapanisertib doses of ≥4 mg (Supplementary Fig. 3). There was a trend toward a dose-dependent PD effect between 2–6 mg sapanisertib; however, no noteworthy differences in PD marker trends were observed at doses ≥6 mg or across the different dosing schedules and dose levels. PD data in tumour tissue were limited due to the small number of samples. For the five patients with paired tumour samples available for review (biopsies at baseline and cycle 1, week 2), the levels of p4EBP1, pS6, pPRAS40 and pNDRG1 decreased after sapanisertib treatment (Supplementary Fig. 4). An integrated PK/PD analysis correlated the plasma concentration of sapanisertib at a single time point with PD findings in skin and demonstrated a concentration-related change in pS6, p4EBP1, pNDRG1 and pPRAS40 (Supplementary Fig. 5).

## Discussion

In this open-label, Phase 1, first-in-human study, the safety profile of sapanisertib was characterised and shown to be manageable and consistent with the toxicity profiles of other mTOR inhibitors. Sapanisertib showed preliminary antitumour activity in patients with RCC (TORC1 inhibitor–naïve and exposed) and in endometrial cancer.

The sapanisertib MTDs were determined as 6 mg QD, 40 mg QW, 9 mg QD × 3dQW and 7 mg QD × 5dQW. In a previous study in advanced haematological malignancies, the sapanisertib MTD for QD dosing was slightly lower (4 mg) and the MTD for QD × 3dQW was the same (9 mg).^25^ Consistent with the lower MTD determined for QD dosing, 5 mg QD (expansion phase) was selected for further investigation, as 6 mg QD was poorly tolerated beyond the DLT evaluation period. DLTs varied across the dosing schedules: maculo-papular rash and hyperglycaemia, considered on-target toxicities for this class of agents, were the most frequently reported DLTs with QD dosing, and stomatitis and asthenia with the QD × 3dQW and QD × 5dQW schedules. Sapanisertib DLTs previously reported in patients with haematologic malignancies, were stomatitis, urticaria, blood creatinine elevation, fatigue, nausea and vomiting with QD dosing, and erythematous rash, fatigue, asthenia, mucosal inflammation and thrombocytopenia with QD × 3dQW dosing.^25^

The safety profile of sapanisertib in this Phase 1 study was generally manageable across all schedules, and tolerability was greater with increased intermittence of dosing. Common AEs related to sapanisertib across all schedules in both phases included hyperglycaemia, nausea and vomiting. Hyperglycaemia is a known side effect of PI3K pathway inhibition reported in other studies investigating dual mTORC inhibitors.^26,27^ Hyperglycaemia was found to be dose-dependent in the QD dosing schedule, occurring more frequently at the higher QW dose. Hyperglycaemia in both phases was generally grade 1–2 in severity, consistent with episodes previously reported,^25^ and easily controlled with metformin therapy. Otherwise, there were no consistent dose-dependent trends in AEs related to sapanisertib among the schedules, and AEs reported for sapanisertib 5 mg QD and 30 mg QW in the expansion phase were generally manageable. In the expansion phase, the sapanisertib dose for QW dosing was reduced from 40 mg (MTD determined during dose escalation) to 30 mg due to high rates of nausea and vomiting.

Preliminary antitumour activity was observed with sapanisertib across different schedules in patients with RCC and endometrial cancer. One patient receiving sapanisertib 40 mg QW had a CR and nine patients achieved PR, with response maintained in two patients for >32 months and four for >16 months. This is consistent with efficacy reported in other phase 1/2 studies of dual TORC1/2 kinase inhibitors in patients with advanced malignancies,^25,27–29^ and the ORR of 22% reported in patients with RCC naïve to a TORC1 inhibitor compares favourably to ORRs observed with rapalogs in patients with RCC (ORR 8.6% with temsirolimus^8^ and 1–5% with everolimus).^9,7,30,31^ However, a recent Phase 2 study of sapanisertib 30 mg QW versus everolimus 10 mg QD in patients with refractory clear-cell RCC reported no responses with single-agent sapanisertib versus an ORR of 13% with everolimus (NCT02724020).^32^

The PK profile showed that single doses of oral sapanisertib were rapidly absorbed and concentrations increased in a dose-dependent manner between 2 and 40 mg; the PK of sapanisertib was time-linear and supported the use of multiple dosing. Following repeat dosing, sapanisertib did not accumulate in plasma to any appreciable extent in any of the dosing schedules. Ghobrial et al.^25^ reported a similar PK profile, supporting the administration of both daily and intermittent schedules. Therefore, QW and QD dosing schedules were selected for expansion based on their apparent similar preliminary antitumour activity and better tolerability. PD findings demonstrated treatment-related reductions in TORC1/2 biomarkers (p4EBP1, pS6, pPRAS40 and pNDRG1), which supports dual TORC1/2 inhibition of sapanisertib in doses ≥4 mg.

In conclusion, sapanisertib had a manageable safety profile across the various schedules studied. Recommended Phase 2 doses, based on the data presented here, include 30 mg QW and 5 mg QD. Preliminary antitumour activity was observed in RCC and endometrial cancer. Phase 2 studies of sapanisertib (QW dosing) in patients with previously treated, metastatic clear-cell RCC (NCT03097328) and endometrial cancer (NCT02725268) have completed enrolment. Findings from these studies and those presented here contribute to sapanisertib dosing decisions in ongoing Phase 1/2 studies as a single agent or in combination with standard-of-care therapy in multiple malignancies.

## Supplementary information

Voss supplementary material

## Data Availability

The datasets, including the redacted study protocol, redacted statistical analysis plan and individual participants data supporting the results reported in this article, will be made available within three months from initial request, to researchers who provide a methodologically sound proposal. The data will be provided after its de-identification, in compliance with applicable privacy laws, data protection and requirements for consent and anonymisation.

## References

[1] Porta C, Paglino C, Mosca A (2014). Targeting PI3K/Akt/mTOR signaling in cancer. Front. Oncol..

[2] Zoncu R, Efeyan A, Sabatini DM (2011). mTOR: from growth signal integration to cancer, diabetes and ageing. Nat. Rev. Mol. Cell Biol..

[3] Duran I, Lambea J, Maroto P, Gonzalez-Larriba JL, Flores L, Granados-Principal S (2017). Resistance to targeted therapies in renal cancer: the importance of changing the mechanism of action. Target. Oncol..

[4] Moschetta M, Reale A, Marasco C, Vacca A, Carratu MR (2014). Therapeutic targeting of the mTOR-signalling pathway in cancer: benefits and limitations. Br. J. Pharm..

[5] Yardley DA, Noguchi S, Pritchard KI, Burris HA, Baselga J, Gnant M (2013). Everolimus plus exemestane in postmenopausal patients with HR(+) breast cancer: BOLERO-2 final progression-free survival analysis. Adv. Ther..

[6] Yao JC, Fazio N, Singh S, Buzzoni R, Carnaghi C, Wolin E (2016). Everolimus for the treatment of advanced, non-functional neuroendocrine tumours of the lung or gastrointestinal tract (RADIANT-4): a randomised, placebo-controlled, phase 3 study. Lancet.

[7] Motzer RJ, Escudier B, Oudard S, Hutson TE, Porta C, Bracarda S (2008). Efficacy of everolimus in advanced renal cell carcinoma: a double-blind, randomised, placebo-controlled phase III trial. Lancet.

[8] Hudes G, Carducci M, Tomczak P, Dutcher J, Figlin R, Kapoor A (2007). Temsirolimus, interferon alfa, or both for advanced renal-cell carcinoma. N. Engl. J. Med..

[9] AFINITOR^®^ (everolimus) Prescribing Information. April 2018 revision.

[10] Torisel^®^ (temsorolimus) Prescribing Information. February 2015 revision.

[11] Kajiwara, M., Masuda, S. Role of mTOR inhibitors in kidney disease. *Int. J. Mol. Sci.* **17**, 1–12 10.3390/ijms17060975 (2016). 10.3390/ijms17060975 PMC4926507 27338360

[12] O’Reilly KE, Rojo F, She QB, Solit D, Mills GB, Smith D (2006). mTOR inhibition induces upstream receptor tyrosine kinase signaling and activates Akt. Cancer Res..

[13] Guertin DA, Stevens DM, Saitoh M, Kinkel S, Crosby K, Sheen JH (2009). mTOR complex 2 is required for the development of prostate cancer induced by Pten loss in mice. Cancer Cell.

[14] Zhou H, Luo Y, Huang S (2010). Updates of mTOR inhibitors. Anticancer Agents Med. Chem..

[15] Bhagwat SV, Gokhale PC, Crew AP, Cooke A, Yao Y, Mantis C (2011). Preclinical characterization of OSI-027, a potent and selective inhibitor of mTORC1 and mTORC2: distinct from rapamycin. Mol. Cancer Ther..

[16] Janes MR, Vu C, Mallya S, Shieh MP, Limon JJ, Li LS (2013). Efficacy of the investigational mTOR kinase inhibitor MLN0128/INK128 in models of B-cell acute lymphoblastic leukemia. Leukemia.

[17] Korets SB, Musa F, Curtin J, Blank SV, Schneider RJ (2014). Dual mTORC1/2 inhibition in a preclinical xenograft tumor model of endometrial cancer. Gynecol. Oncol..

[18] Zheng B, Mao JH, Qian L, Zhu H, Gu DH, Pan XD (2015). Pre-clinical evaluation of AZD-2014, a novel mTORC1/2 dual inhibitor, against renal cell carcinoma. Cancer Lett..

[19] Garcia-Garcia C, Ibrahim YH, Serra V, Calvo MT, Guzman M, Grueso J (2012). Dual mTORC1/2 and HER2 blockade results in antitumor activity in preclinical models of breast cancer resistant to anti-HER2 therapy. Clin. Cancer Res..

[20] Gokmen-Polar Y, Liu Y, Toroni RA, Sanders KL, Mehta R, Badve S (2012). Investigational drug MLN0128, a novel TORC1/2 inhibitor, demonstrates potent oral antitumor activity in human breast cancer xenograft models. Breast Cancer Res. Treat..

[21] Hernandez-Prat A, Rodriguez-Vida A, Juanpere-Rodero N, Arpi O, Menendez S, Soria-Jimenez L (2019). Novel oral mTORC1/2 inhibitor TAK-228 has synergistic antitumor effects when combined with paclitaxel or PI3Kalpha inhibitor TAK-117 in preclinical bladder cancer models. Mol. Cancer Res..

[22] Ingels A, Zhao H, Thong AE, Saar M, Valta MP, Nolley R (2014). Preclinical trial of a new dual mTOR inhibitor, MLN0128, using renal cell carcinoma tumorgrafts. Int. J. Cancer.

[23] Kang MH, Reynolds CP, Maris JM, Gorlick R, Kolb EA, Lock R (2014). Initial testing (stage 1) of the investigational mTOR kinase inhibitor MLN0128 by the pediatric preclinical testing program. Pediatr. Blood Cancer.

[24] Eisenhauer EA, Therasse P, Bogaerts J, Schwartz LH, Sargent D, Ford R (2009). New response evaluation criteria in solid tumours: revised RECIST guideline (version 1.1). Eur. J. Cancer.

[25] Ghobrial IM, Siegel DS, Vij R, Berdeja JG, Richardson PG, Neuwirth R (2016). TAK-228 (formerly MLN0128), an investigational oral dual TORC1/2 inhibitor: a phase I dose escalation study in patients with relapsed or refractory multiple myeloma, non‐Hodgkin lymphoma, or Waldenström’s macroglobulinemia. Am. J. Hematol..

[26] Bauer TM, Patel MR, Infante JR (2015). Targeting PI3 kinase in cancer. Pharm. Ther..

[27] Bendell JC, Kelley RK, Shih KC, Grabowsky JA, Bergsland E, Jones S (2015). A phase I dose-escalation study to assess safety, tolerability, pharmacokinetics, and preliminary efficacy of the dual mTORC1/mTORC2 kinase inhibitor CC-223 in patients with advanced solid tumors or multiple myeloma. Cancer.

[28] Basu B, Dean E, Puglisi M, Greystoke A, Ong M, Burke W (2015). First-in-human pharmacokinetic and pharmacodynamic study of the dual m-TORC 1/2 inhibitor AZD2014. Clin. Cancer Res..

[29] Powles T, Wheater M, Din O, Geldart T, Boleti E, Stockdale A (2016). A randomised phase 2 study of AZD2014 versus everolimus in patients with VEGF-refractory metastatic clear cell renal cancer. Eur. Urol..

[30] Choueiri TK, Escudier B, Powles T, Tannir NM, Mainwaring PN, Rini BI (2016). Cabozantinib versus everolimus in advanced renal cell carcinoma (METEOR): final results from a randomised, open-label, phase 3 trial. Lancet Oncol..

[31] Motzer RJ, Escudier B, McDermott DF, George S, Hammers HJ, Srinivas S (2015). Nivolumab versus everolimus in advanced renal-cell carcinoma. N. Engl. J. Med..

[32] Choueiri, T. K., Porta, C., Suárez, C., Hainsworth, J., Voog, E., Duran, I. et al. Randomised phase 2 study of sapanisertib (TAK‑228/MLN0128) ± TAK-117 versus everolimus in patients with VEGF-targeted therapy-refractory metastatic clear cell renal cell carcinoma. (Oral presentation at EIKCS, Dubrovnik, 2019).

