# Supplementary material for: Phase 1 study of mTORC1/2 inhibitor sapanisertib (TAK-228) in advanced solid tumours, with an expansion phase in renal, endometrial or bladder cancer
Source: Br J Cancer. 2020 Sep 11;123(11):1590–8. doi: 10.1038/s41416-020-01041-x (PMC7686313; doi:10.1038/s41416-020-01041-x)
Supplement: Supplementary file 1 — Voss supplementary material [file 41416_2020_1041_MOESM1_ESM.docx]

**SUPPLEMENTARY MATERIALS**

**Results**

**Figures**

**Supplementary Fig. 1** Study duration and best percentage change of tumour size by dosing schedule in patients with (**a**) TORC1 inhibitor (TORC1i)−naïve RCC, (**b**) RCC with TORC1i-failure, (**c**) endometrial cancer, or (**d**) bladder cancer. *CR* complete response, *PD* progressive disease, *PR* partial response, *QD* once daily, *QW* once weekly, *RCC* renal cell carcinoma, *SD* stable disease, *SLD* sum of longest diameters, *TORC1* target of rapamycin complex 1


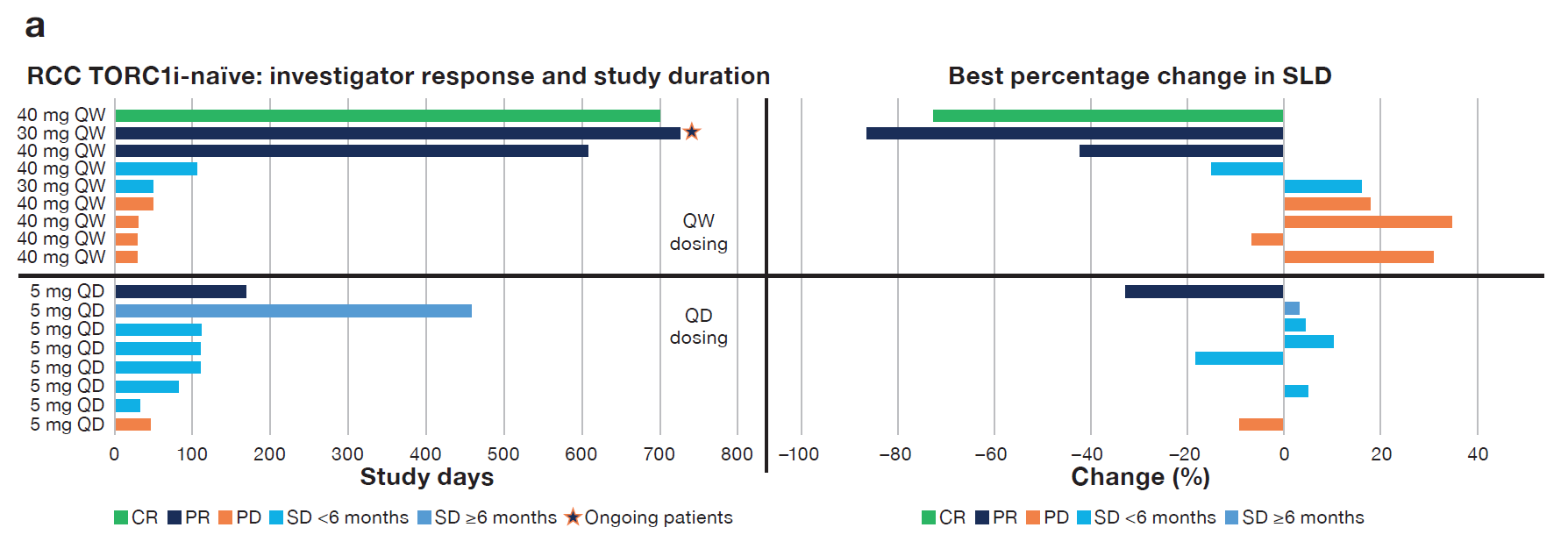


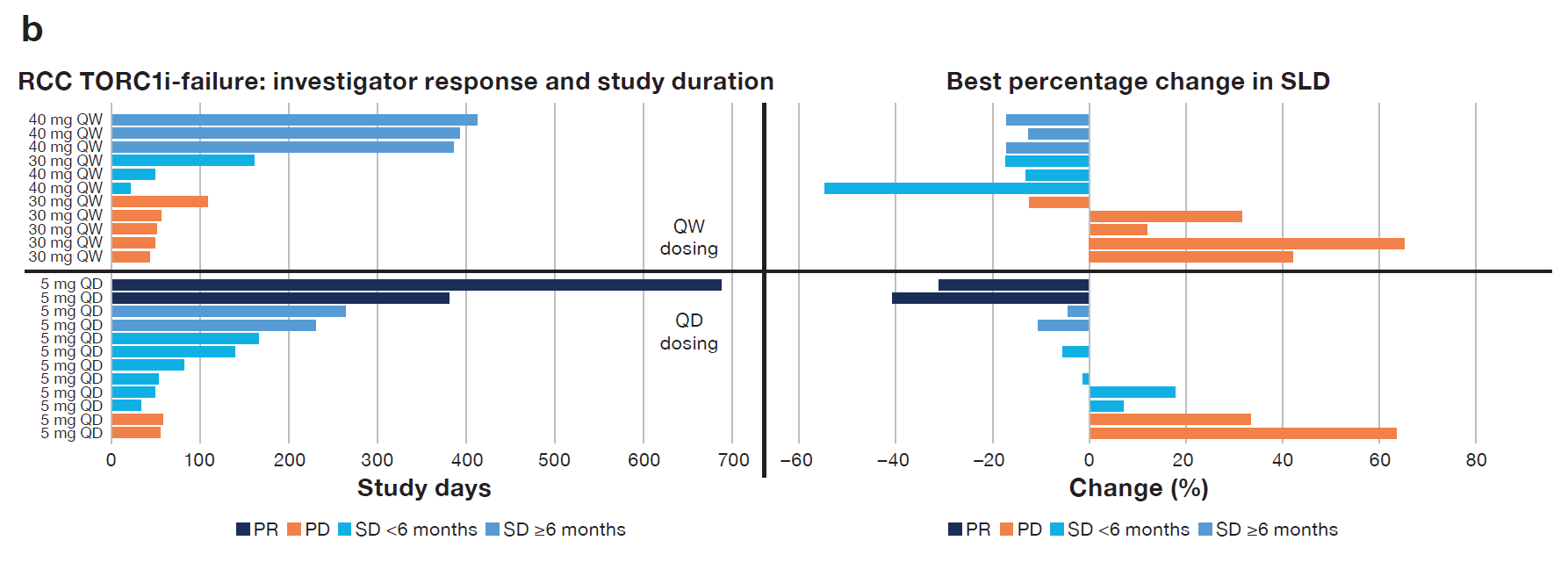


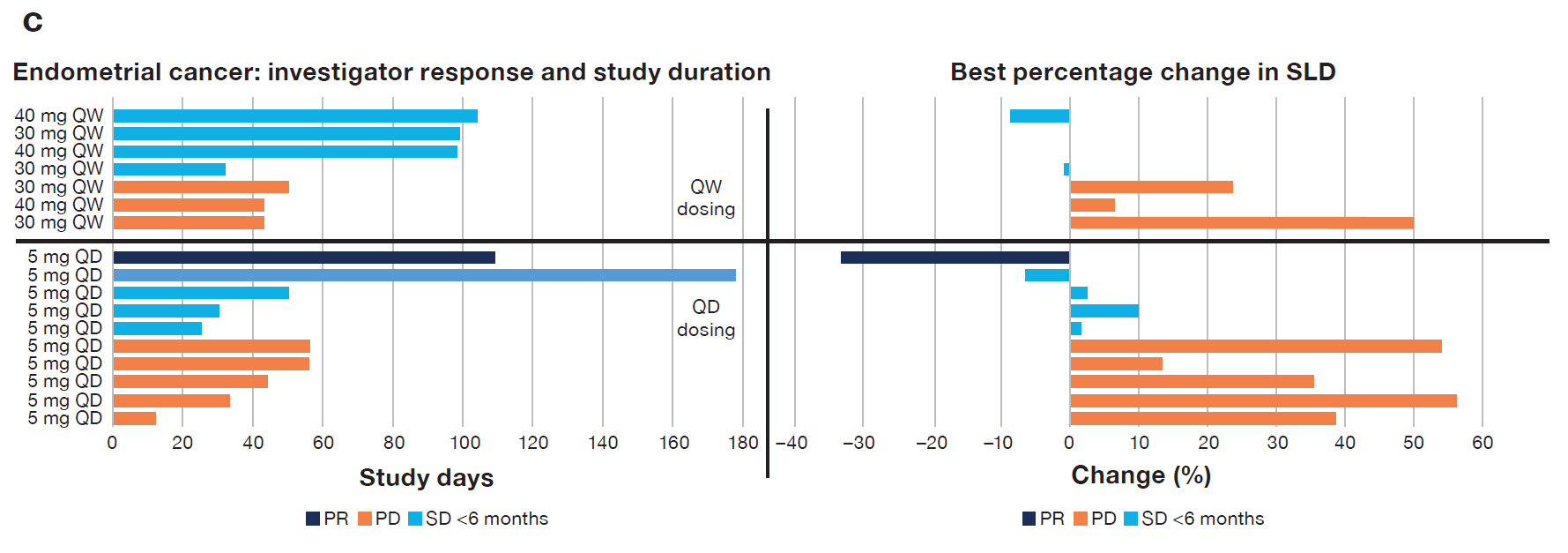


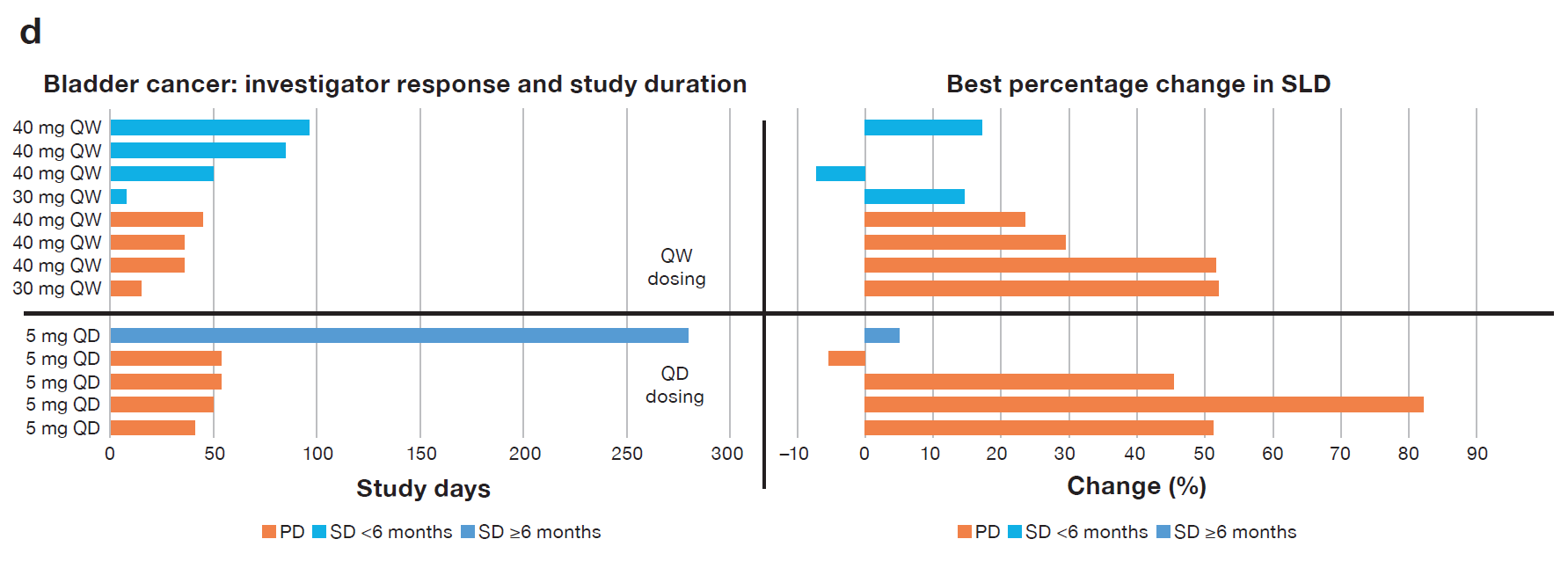


**Supplementary Fig. 2** Mean (SD) plasma concentration–time profiles of single-dose sapanisertib on cycle 1, day 1. Error bars indicate SD. *SD* standard deviation


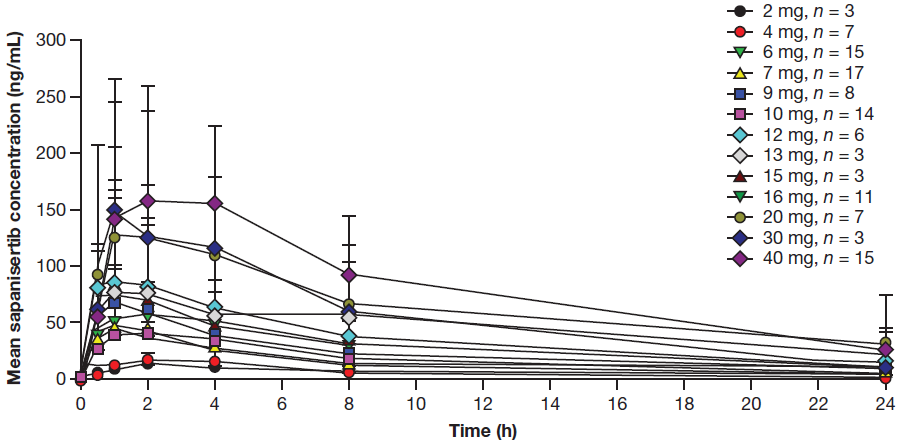


**Supplementary Fig. 3** Pharmacodynamic analysis shows treatment-related inhibition of mammalian target of rapamycin complex 1 (mTORC1/2) biomarkers (**a**) p4EBP1, (**b**) pS6, (**c**) pPRAS40, and (**d**) pNDRG1 in skin 2–4 h post-dose on any dosing day between days 8–15


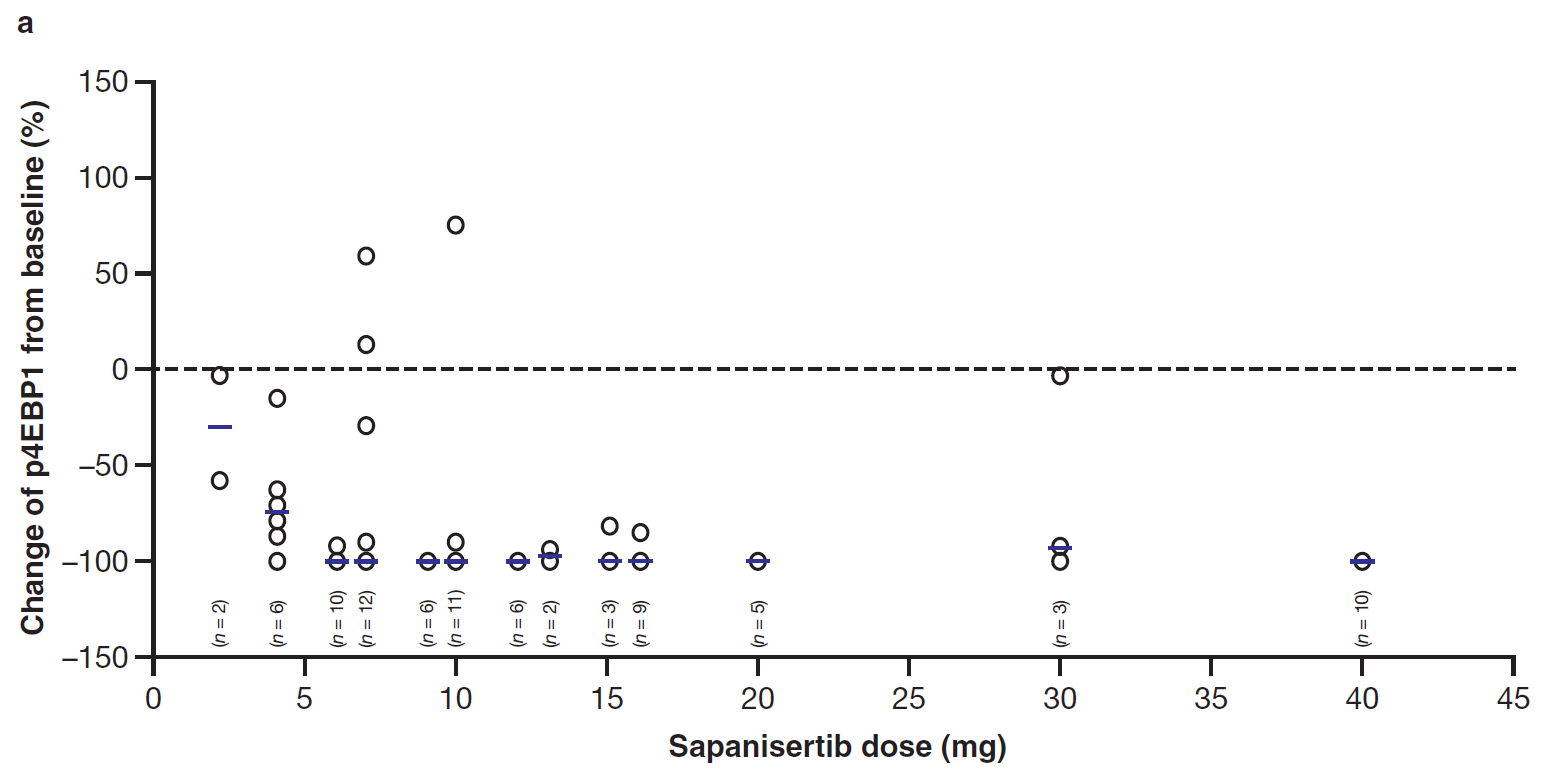


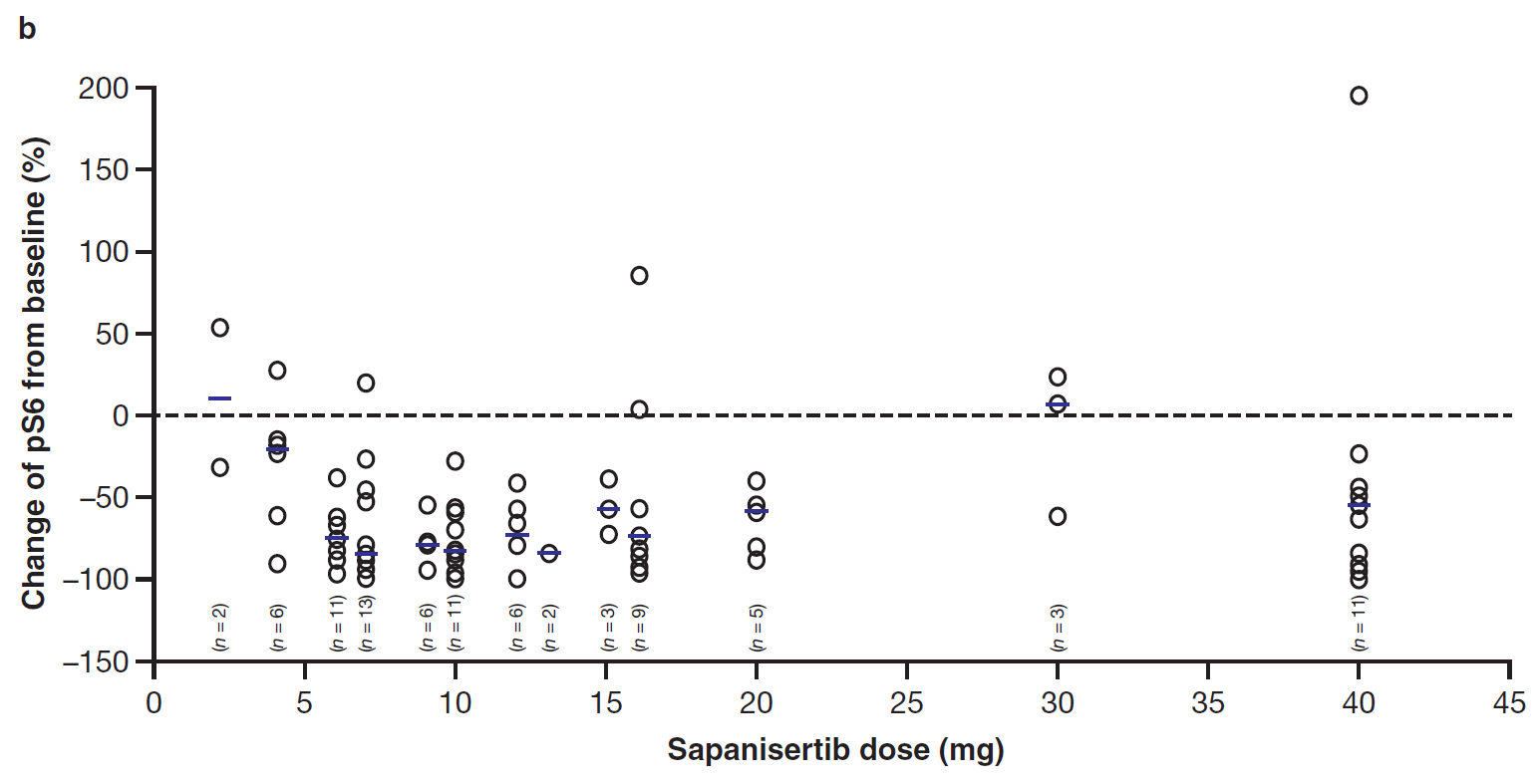


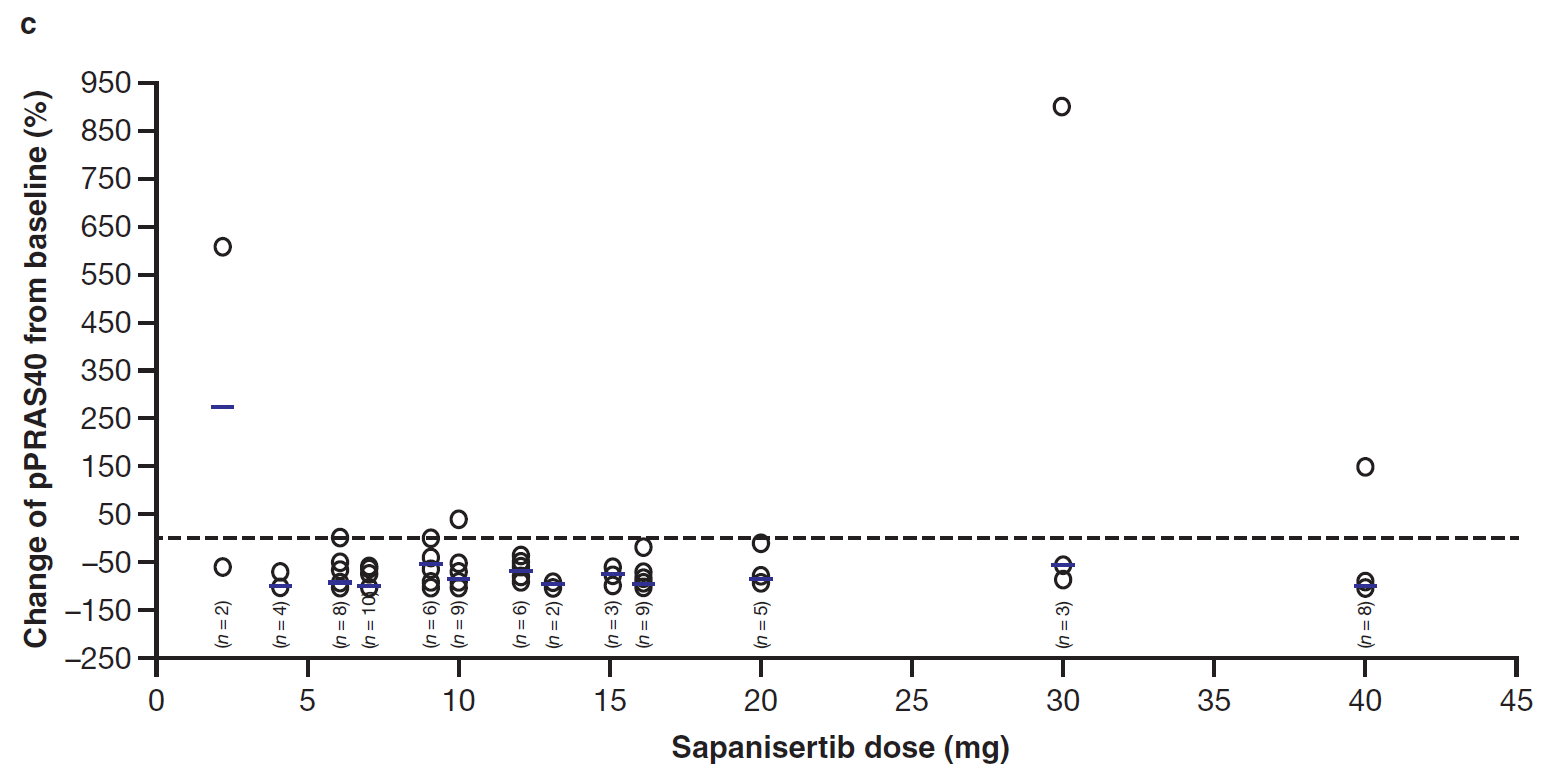


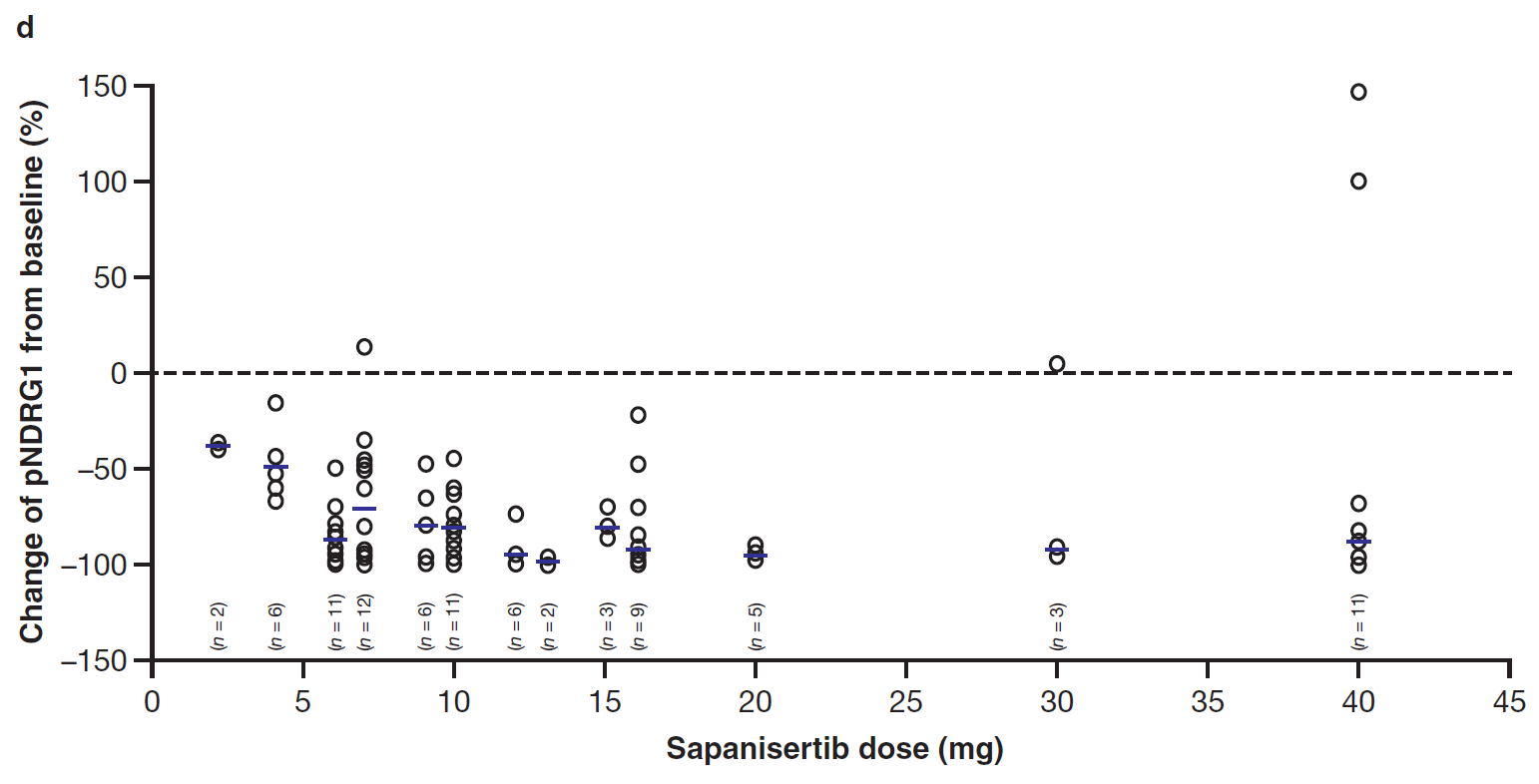


**Supplementary Fig. 4** Percentage change from baseline of pharmacodynamic markers p4EBP1, pS6, pPRAS40, and pNDRG1 in tumour biopsies. *QD* once daily, *QD×3d* QD for 3 days on/4 days off


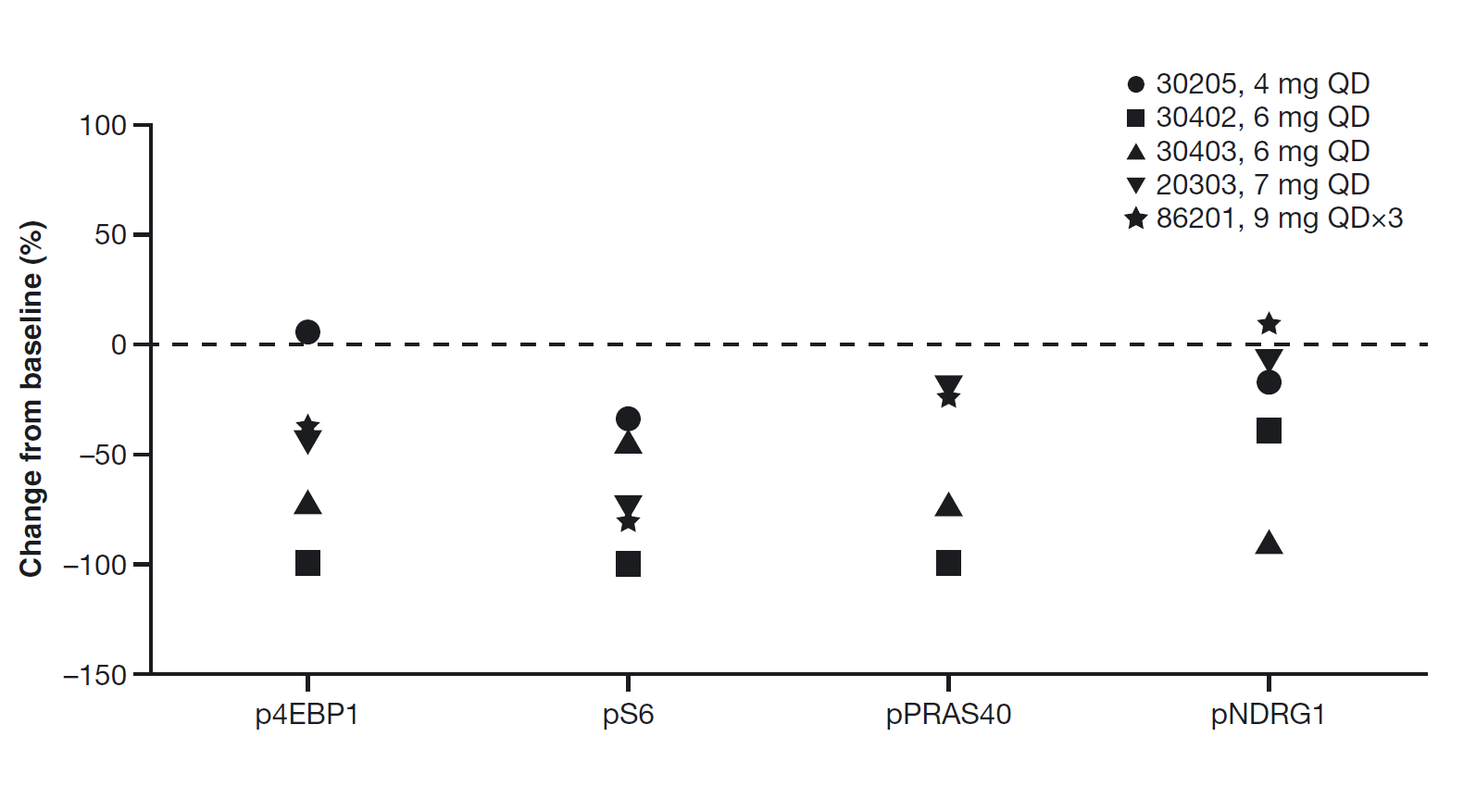


**Supplementary Fig. 5** Sapanisertib concentration versus target of rapamycin complex 1 (TORC1) biomarkers ([**a**] pS6 and [**b**] p4EBP1) and TORC2 biomarkers ([**c**] pNRDG1 and [**d**] pPRAS40)

**
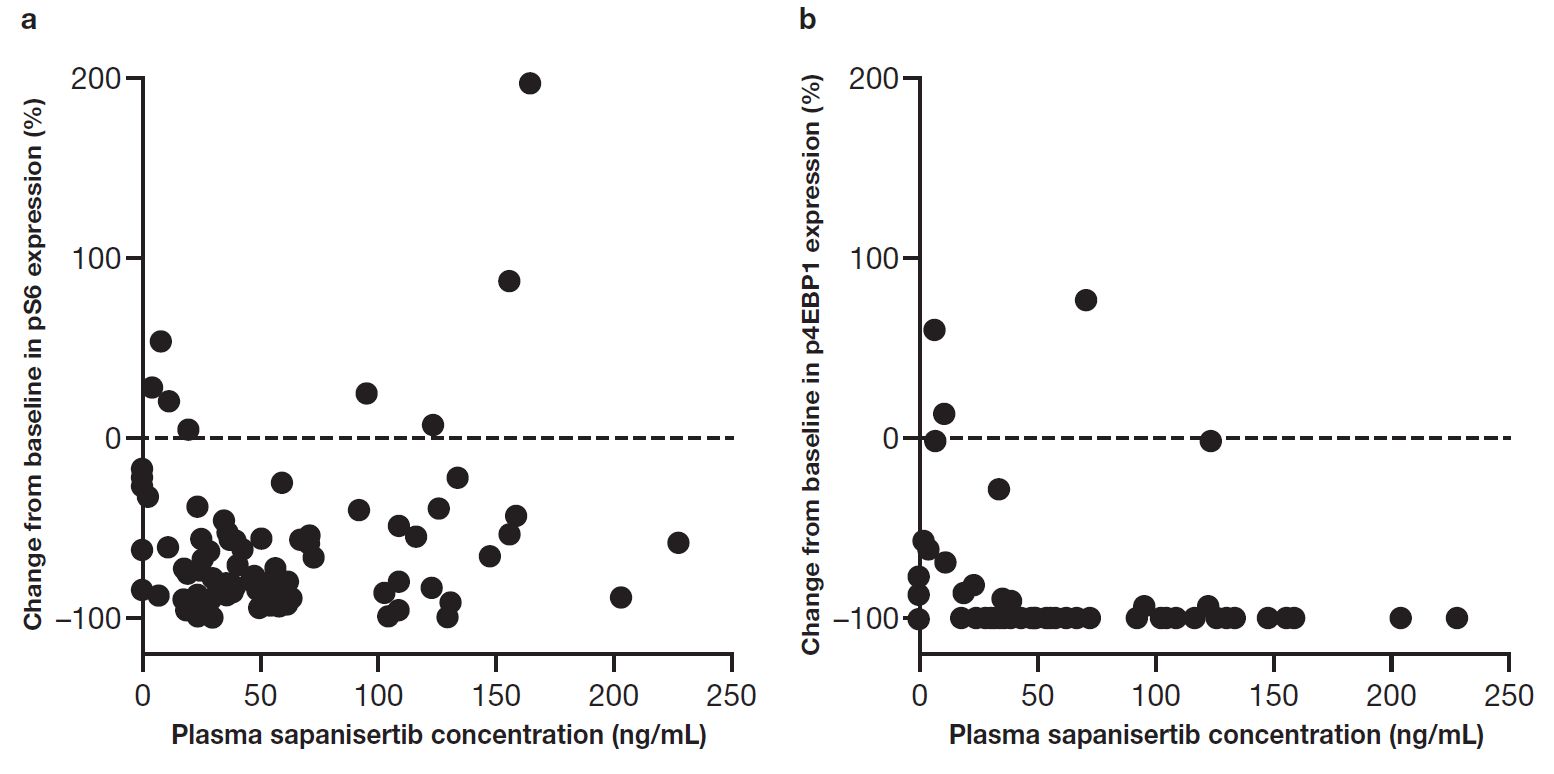
**

**
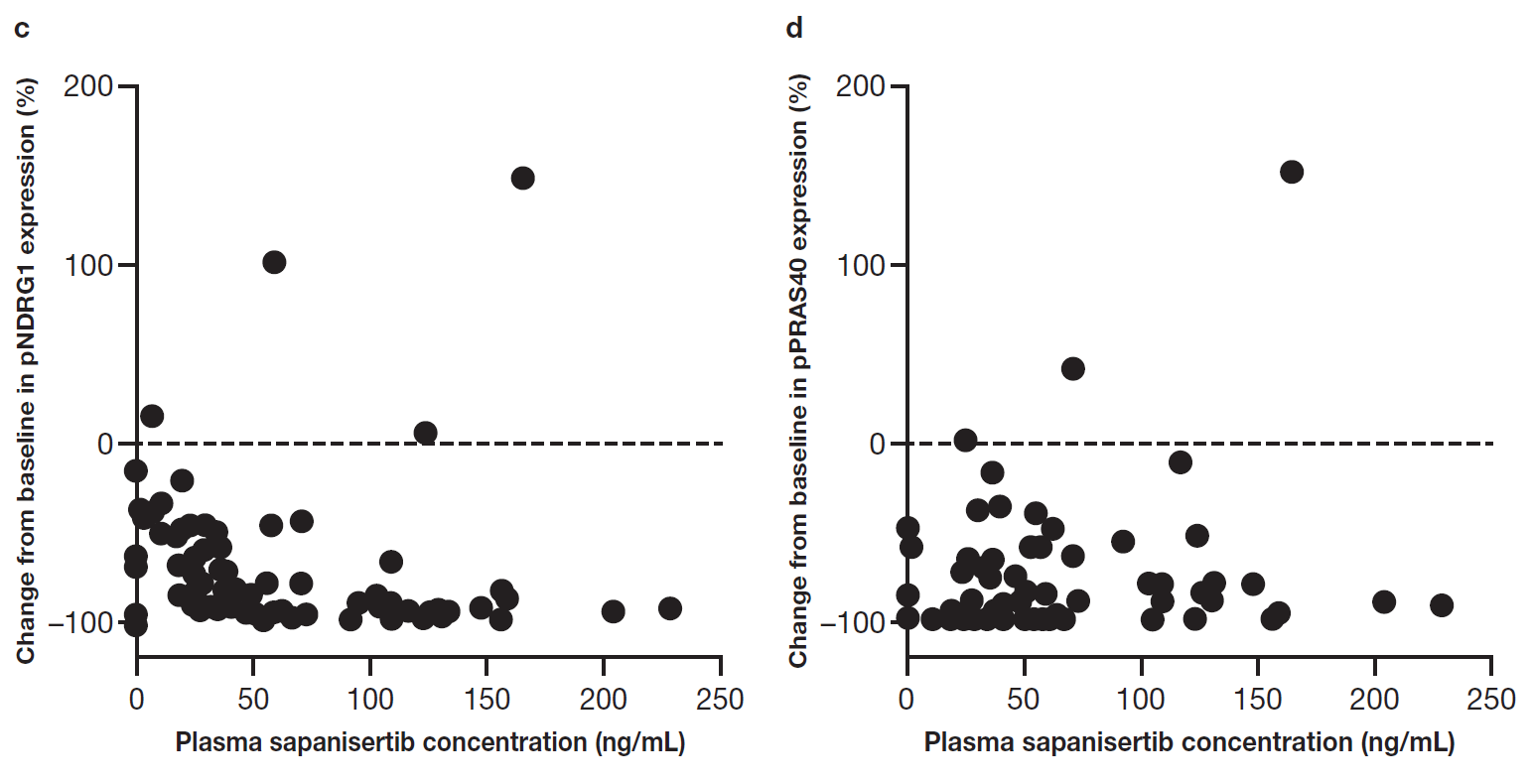
**

**Supplementary Table 1.** Summary of safety profiles of sapanisertib by dosing schedule

|  | Sapanisertib dosing schedule | | | | | | | | |
| --- | --- | --- | --- | --- | --- | --- | --- | --- | --- |
|  | Dose-escalation phase | | | | | Expansion phase | | | |
| AE, *n* (%) | QD 2–7 mg (*n* = 31) | QD×3dQW 6–20 mg (*n* = 33) | QD×5dQW 7–13 mg (*n* = 22) | QW 7–40 mg (*n* = 30) | Total (*n* = 116) | QD 5 mg (*n* = 39) | QW 30 mg (*n* = 17) | QW 40 mg (*n* = 26) | Total (*n* = 82) |
| Any AE | 31 (100) | 33 (100) | 22 (100) | 30 (100) | 116 (100) | 39 (100) | 17 (100) | 26 (100) | 82 (100) |
| Any treatment-related AE | 31 (100) | 32 (97) | 21 (95) | 28 (93) | 112 (97) | 39 (100) | 17 (100) | 26 (100) | 82 (100) |
| Any grade ≥3 AE | 19 (61) | 25 (76) | 14 (64) | 18 (60) | 76 (66) | 30 (77) | 9 (53) | 19 (73) | 58 (71) |
| Any treatment-related grade ≥3 AE | 14 (45) | 19 (58) | 8 (36) | 8 (27) | 49 (42) | 18 (46) | 6 (35) | 14 (54) | 38 (46) |
| Any AE resulting in treatment discontinuation | 11 (35) | 7 (21) | 6 (27) | 4 (13) | 28 (24) | 7 (18) | 3 (18) | 2 (8) | 12 (15) |
| Any AE resulting in dose modification/interruption | 22 (71) | 21 (64) | 12 (55) | 13 (43) | 68 (59) | 27 (69) | 8 (47) | 20 (77) | 55 (67) |
| Any SAE | 13 (42) | 17 (52) | 10 (45) | 10 (33) | 50 (43) | 19 (49) | 6 (35) | 9 (35) | 34 (41) |
| Any treatment-related SAE | 4 (13) | 9 (27) | 4 (18) | 0 | 17 (15) | 5 (13) | 3 (18) | 4 (15) | 12 (15) |
| On-study deaths | 1 (3) | 2 (6) | 0 | 1 (3) | 4 (3) | 0 | 2 (12) | 1 (4) | 3 (4) |
| Median number of cycles, median (range) | 2 (1–13) | 2 (1–33) | 2 (1–40) | 2 (1–58) | 2 (1–58) | 2 (1–25) | 2 (1–26) | 2 (1–25) | 2 (1–26) |

*AE* adverse event, *QD* once daily, *QD×3dQW* once daily for 3 days on and 4 days off each week, *QD×5dQW* once daily for 5 days on and 2 days off each week, *QW* once weekly, *SAE* serious AE

**Supplementary Table 2.** Treatment-related grade ≥3 adverse events (AEs) reported in ≥2 patients

|  | Sapanisertib regimen | | | | | | | | |
| --- | --- | --- | --- | --- | --- | --- | --- | --- | --- |
|  | Dose-escalation phase | | | | | Expansion phase | | | |
| AE, *n* (%) | QD 2–7 mg  (*n* = 31) | QD×5dQW 7–13 mg (*n* = 22) | QD×3dQW 6–20 mg (*n* = 33) | QW 7–40 mg (*n* = 30) | Total (*n* = 116) | QD 5 mg (*n* = 39) | QW 30 mg (*n* = 17) | QW 40 mg (*n* = 26) | Total (*n* = 82) |
| Hyperglycaemia | 6 (19) | 1 (5) | 7 (21) | 2 (7) | 16 (14) | 5 (13) | 2 (12) | 6 (23) | 13 (16) |
| Asthenia | 1 (3) | 3 (14) | 4 (12) | 1 (3) | 9 (8) | 1 (3) | 0 (0) | 1 (4) | 2 (2) |
| Stomatitis | 0 (0) | 3 (14) | 5 (15) | 0 (0) | 8 (7) | 3 (8) | 0 (0) | 1 (4) | 4 (5) |
| Lymphopenia | 2 (6) | 0 (0) | 2 (6) | 1 (3) | 5 (4) | 0 (0) | 0 (0) | 0 (0) | 0 (0) |
| Fatigue | 0 (0) | 2 (9) | 0 (0) | 2 (7) | 4 (3) | 4 (10) | 2 (12) | 4 (15) | 10 (12) |
| Hypophosphatemia | 0 (0) | 0 (0) | 3 (9) | 0 (0) | 3 (3) | 3 (8) | 1 (6) | 2 (8) | 6 (7) |
| Nausea | 0 (0) | 1 (5) | 2 (6) | 0 (0) | 3 (3) | 1 (3) | 1 (6) | 2 (8) | 4 (5) |
| Pruritus generalised | 2 (6) | 0 (0) | 1 (3) | 0 (0) | 3 (3) | 1 (3) | 0 (0) | 0 (0) | 1 (1) |
| Rash maculo-papular | 2 (6) | 1 (5) | 0 (0) | 0 (0) | 3 (3) | 3 (8) | 0 (0) | 0 (0) | 3 (4) |
| Rash | 2 (6) | 0 (0) | 0 (0) | 0 (0) | 2 (2) | 0 (0) | 0 (0) | 0 (0) | 0 (0) |
| Thrombocytopenia | 2 (6) | 0 (0) | 0 (0) | 0 (0) | 2 (2) | 2 (5) | 1 (6) | 1 (4) | 4 (5) |
| Diarrhoea | 1 (3) | 0 (0) | 0 (0) | 1 (3) | 2 (2) | 2 (5) | 0 (0) | 1 (4) | 3 (4) |
| Dehydration | 0 (0) | 0 (0) | 1 (3) | 0 (0) | 1 (1) | 0 (0) | 0 (0) | 2 (8) | 2 (2) |

QD once daily, QW once weekly, QD×3dQW once daily for 3 days on and 4 days off each week, QD×5dQW once daily for 5 days on and 2 days off each week.

**Supplementary Table 3.** All-grade adverse events (AEs) of any cause by preferred term reported in ≥20% of patients by dosing schedule in the expansion phase

|  | Sapanisertib regimen | | | |
| --- | --- | --- | --- | --- |
|  | Expansion phase | | | |
| AE, *n* (%) | QD 5 mg (*n* = 39) | QW 30 mg (*n* = 17) | QW 40 mg (*n* = 26) | Total (*n* = 82) |
| Fatigue | 25 (64) | 11 (65) | 24 (92) | 60 (73) |
| Nausea | 21 (54) | 13 (76) | 20 (77) | 54 (66) |
| Hyperglycaemia | 18 (46) | 12 (71) | 20 (77) | 50 (61) |
| Vomiting | 13 (33) | 10 (59) | 20 (77) | 43 (52) |
| Decreased appetite | 20 (51) | 7 (41) | 14 (54) | 41 (50) |
| Diarrhoea | 20 (51) | 8 (47) | 13 (50) | 41 (50) |
| Stomatitis | 19 (49) | 9 (53) | 12 (46) | 40 (49) |
| Constipation | 10 (26) | 8 (47) | 10 (38) | 28 (34) |
| Rash maculo-papular | 17 (44) | 3 (18) | 4 (15) | 24 (29) |
| Cough | 9 (23) | 5 (29) | 8 (31) | 22 (27) |
| Anaemia | 6 (15) | 2 (12) | 11 (42) | 19 (23) |
| Pruritus generalised | 12 (31) | 4 (24) | 3 (12) | 19 (23) |
| Abdominal pain | 7 (18) | 6 (35) | 4 (15) | 17 (21) |
| Dehydration | 6 (15) | 4 (24) | 7 (27) | 17 (21) |
| Dysgeusia | 10 (26) | 4 (24) | 3 (12) | 17 (21) |
| Dry mouth | 5 (13) | 3 (18) | 7 (27) | 15 (18) |
| Oedema peripheral | 9 (23) | 1 (6) | 5 (19) | 15 (18) |
| Pruritus | 10 (26) | 1 (6) | 4 (15) | 15 (18) |
| Urinary tract infection | 6 (15) | 1 (6) | 8 (31) | 15 (18) |
| Weight decreased | 6 (15) | 2 (12) | 7 (27) | 15 (18) |
| Blood creatinine increased | 5 (13) | 4 (24) | 5 (19) | 14 (17) |
| Pyrexia | 8 (21) | 1 (6) | 5 (19) | 14 (17) |
| Oropharyngeal pain | 3 (8) | 1 (6) | 9 (35) | 13 (16) |
| Dizziness | 9 (23) | 0 (0) | 3 (12) | 12 (15) |
| Headache | 4 (10) | 2 (12) | 6 (23) | 12 (15) |
| Arthralgia | 2 (5) | 2 (12) | 7 (27) | 11 (13) |
| Depression | 1 (3) | 4 (24) | 3 (12) | 8 (10) |

*QD* once daily, *QW* once weekly

**Supplementary Table 4.** Grade ≥3 adverse events (AEs) of any cause reported in ≥2 patients in the expansion phase

|  | Sapanisertib regimen | | | |
| --- | --- | --- | --- | --- |
|  | Expansion phase | | | |
| Grade ≥3 AE, *n* (%) | QD 5 mg (*n* = 39) | QW 30 mg (*n* = 17) | QW 40 mg (*n* = 26) | Total (*n* = 82) |
| Hyperglycaemia | 5 (13) | 2 (12) | 6 (23) | 13 (16) |
| Fatigue | 5 (13) | 2 (12) | 4 (15) | 11 (13) |
| Hypophosphatemia | 4 (10) | 2 (12) | 2 (8) | 8 (10) |
| Anaemia | 1 (3) | 1 (6) | 3 (12) | 5 (6) |
| Nausea | 1 (3) | 2 (12) | 2 (8) | 5 (6) |
| Acute kidney injury | 2 (5) | 0 (0) | 2 (8) | 4 (5) |
| Rash maculo-papular | 3 (8) | 0 (0) | 1 (4) | 4 (5) |
| Stomatitis | 3 (8) | 0 (0) | 1 (4) | 4 (5) |
| Thrombocytopenia | 2 (5) | 1 (6) | 1 (4) | 4 (5) |
| Abdominal pain | 3 (8) | 0 (0) | 0 (0) | 3 (4) |
| Asthenia | 1 (3) | 0 (0) | 2 (8) | 3 (4) |
| Dehydration | 0 (0) | 0 (0) | 3 (12) | 3 (4) |
| Diarrhoea | 2 (5) | 0 (0) | 1 (4) | 3 (4) |
| Hyponatremia | 1 (3) | 1 (6) | 1 (4) | 3 (4) |
| Pain in extremity | 0 (0) | 2 (12) | 1 (4) | 3 (4) |
| Vomiting | 1 (3) | 1 (6) | 1 (4) | 3 (4) |
| Constipation | 0 (0) | 1 (6) | 1 (4) | 2 (2) |
| Dyspnoea | 2 (5) | 0 (0) | 0 (0) | 2 (2) |
| Haematuria | 2 (5) | 0 (0) | 0 (0) | 2 (2) |
| Hyperkalaemia | 2 (5) | 0 (0) | 0 (0) | 2 (2) |
| Hypertension | 1 (3) | 0 (0) | 1 (4) | 2 (2) |
| Hypokalaemia | 1 (3) | 1 (6) | 0 (0) | 2 (2) |
| Lymphopenia | 1 (3) | 0 (0) | 1 (4) | 2 (2) |
| Musculoskeletal chest pain | 1 (3) | 0 (0) | 1 (4) | 2 (2) |
| Pneumonia | 2 (5) | 0 (0) | 0 (0) | 2 (2) |
| Sepsis | 1 (3) | 1 (6) | 0 (0) | 2 (2) |
| Transitional cell carcinoma | 0 (0) | 1 (6) | 1 (4) | 2 (2) |

*QD* once daily, *QW* once weekly

**Supplementary Table 5.** Sapanisertib plasma pharmacokinetic parameters during (**a**) cycle 1, day 1 and (**b**) cycle 2, day 1

**a**

| Dose level | T_max_ (h), median | C_max_ (ng/mL) [%CV] | AUC_0–last_ (ng*h/mL)  [%CV] | AUC_0–24h_ (ng*h/mL)  [%CV] | t_½_ (h) |
| --- | --- | --- | --- | --- | --- |
| 2 mg | 2.0 | 13.5 (16.7) | 65.4 (NA) | NA | NA |
| 4 mg | 2.0 | 19.1 (40.1) | 63.2 (32.5) | 178.4 (28.7) | 7.1 |
| 6 mg | 1.0 | 50.8 (45.5) | 124.3 (NA) | 354.3 (26.0) | 6.8 |
| 7 mg | 1.5 | 46.7 (62.1) | NA (NA) | 327.3 (33.0) | 6.9 |
| 9 mg | 1.1 | 75.9 (35.5) | 207.3 (NA) | 595.9 (25.8) | 7.5 |
| 10 mg | 2.8 | 48.4 (54.9) | 153.1 (NA) | 341.8 (60.1) | 6.4 |
| 12 mg | 2.0 | 99.5 (81.9) | NA | 730.2 (43.5) | 7.4 |
| 13 mg | 2.1 | 93.6 (63.0) | NA | 952.6 (59.6) | 8.7 |
| 15 mg | 2.0 | 56.7 (100.8) | NA | 517.8 (86.5) | 9.4 |
| 16 mg | 2.1 | 66.7 (68.5) | 124.9 (NA) | 688.7 (29.1) | 7.0 |
| 20 mg | 2.1 | 154.1 (45.0) | NA | 1262.8 (69.3) | 6.5 |
| 30 mg | 1.0 | 161.8 (45.8) | NA | 1076.8 (66.3) | 5.9 |
| 40 mg | 2.4 | 172.4 (48.6) | NA | 1639.5 (48.5) | 7.6 |

*%CV* percentage coefficient of variation, AUC_0–24h_ area under the curve from time zero to 24 hours post-dose, AUC_0–last_ area under the curve from time zero to time of last quantifiable concentration, *NA* not applicable, *t_½_* terminal disposition phase half-life, *T_max_* time of first occurrence of C_max_

**b**

| Dose level | T_max_ (h), median | C_max_ (ng/mL) [%CV] | AUC_0–last_ (ng*h/mL)  [%CV] | AUC_0–24h_ (ng*h/mL)  [%CV] | t_½_ (h) |
| --- | --- | --- | --- | --- | --- |
| QD Dosing Schedule |  |  |  |  |  |
| 2 mg | 2.0 | 15.6 (101.2) | NA | 185.0 (63.5) | 8.1 (NA) |
| 4 mg | 3.8 | 20.3 (117.4) | 61.9 (NA) | 281.4 (49.8) | 10.3 (1.24) |
| 6 mg | 2.0 | 36.9 (48.5) | NA | 327.0 (22.6) | 7.3 (1.00) |
| 7 mg | 4.0 | 51.3 (91.9) | NA | 350.6 (52.0) | 5.63 (2.02) |
| QW Dosing Schedule |  |  |  |  |  |
| 7 mg | 1 | 65.8 (NA) | NA | 350.8 (NA) | 5.6 (NA) |
| 10 mg | 2.9 | 36.2 (NA) | NA | NA | NA |
| 15 mg | 2.0 | 64.9 (30.1) | NA | 604.4 (33.6) | NA |
| 20 mg | 4.0 | NA | NA | NA | NA |
| 30 mg | 4.0 | 132.8 (48.4) | NA | 1120.9 (55.55) | 5.7 (NA) |
| 40 mg | 2.0 | 231.7 (40.7) | NA | 2222.1 (55.5) | 6.4 (1.86) |
| QDx3dQW Dosing Schedule |  |  |  |  |  |
| 6 mg | 4.0 | 59.2 (20.7) | NA | 777.4 (NA) | 9.1 (NA) |
| 9 mg | 2.1 | 83.1 (34.4) | NA | 706.6 (33.1) | 6.9 (0.78) |
| 12 mg | 3.7 | 113.3 (22.0) | NA | 707.2 (NA) | 4.9 (NA) |
| 16 mg | 2.1 | 92.1 (75.4) | NA | 744.3 (56.3) | 6.6 (2.29) |
| 20 mg | 1.0 | NA | NA | NA | NA |
| QDx5dQW Dosing Schedule |  |  |  |  |  |
| 7 mg | 1.5 | 49.7 (75.2) | NA | 362.5 (45.5) | 6.7 (0.49) |
| 10 mg | 2.0 | 57.0 (33.9) | NA | 366.4 (6.1) | 5.7 (1.30) |
| 13 mg | 2.0 | NA | NA | NA | NA |

*%CV* percentage coefficient of variation, AUC_0–24h_ area under the curve from time zero to 24 hours post-dose, AUC_0–last_ area under the curve from time zero to time of last quantifiable concentration, *NA* not applicable, *t_½_* terminal disposition phase half-life, *T_max_* time of first occurrence of C_max_

# Supplementary Information: Ethics approval and consent to participate

**List of institutional review boards and independent ethics committees**

- Alpha Independent Review Board, 1001 Avenida Pico, C-497, San Clemente, CA 93673, USA
- Cedars-Sinai Medical Center Institutional Review Board, Office of Research Compliance, 8383 Wilshire Blvd., Suite 742, Beverly Hills, CA 90211, USA
- Cleveland Clinic Institutional Review Board, 9500 Euclid Avenue, OS-1, Cleveland, OH 44195, USA
- Comite Etico de Investigacion Clinica, Hospital Clinico, Universtario de Valencia, Pabellion B, 1st Planta, Avenida Blasco Ibanez, 17, 46010 Valencia, Spain
- Comite Etico de Investigacion Clinica, Hospital de la Vall d’Hebron, Passeig de la Vall d’Hebron, 119-129, 08035 Barcelona, Spain
- Henry Ford Health System, Research Administration, One Ford Place-2F, Detroit, MI USA 48202
- Indiana University Institutional Review Board, Office of Research Administration, 980 Indiana Avenue, Indianapolis, IN 46202, USA
- IntegReview Ethical Review Board, 3001 S. Lamar Blvd., Suite 210, Austin, TX 78704, USA
- Memorial Sloan Kettering Cancer Center Institutional Review Board B, 1275 York Avenue, New York, NY 10065, USA
- Office for Human Research Studies, 450 Brookline Avenue, OS229, Boston, MA 02215, USA
- Roswell Park Cancer Institutional Review Board, Elm & Carlton Streets, Buffalo, NY 14263, USA
- University of Miami Human Subjects Research Office, 1500 NW 12th Avenue, Suite I002, Miami, FL 33136, USA
- University of Michigan Medical School Institutional Review Board, 2800 Plymouth Road, Building 520, Room 3214, Ann Arbor, MI 48109-2800, USA
- Vanderbilt University Institutional Review Board, 1313 21st Avenue South, 504 Oxford House, Nashville, TN 37232-4315, USA

# 
